# Transcriptome analysis of *Polygonatum cyrtonema* Hua: identification of genes involved in polysaccharide biosynthesis

**DOI:** 10.1186/s13007-019-0441-9

**Published:** 2019-06-26

**Authors:** Chenkai Wang, Daiyin Peng, Jinhang Zhu, Derui Zhao, Yuanyuan Shi, Shengxiang Zhang, Kelong Ma, Jiawen Wu, Luqi Huang

**Affiliations:** 1 Anhui University of Chinese Medicine and Anhui Academy of Chinese Medicine, Hefei, 230038 China; 2 Anhui Medical University, Hefei, 230032 China; 3 Key Laboratory of Xin’an Medicine, Ministry of Education, Anhui University of Chinese Medicine, Hefei, 230038 China; 4 Clinical College of Integrated Traditional Chinese and Western Medicine, Anhui University of Chinese Medicine, Hefei, China; 5 Synergetic Innovation Center of Anhui Authentic Chinese Medicine Quality Improvement, Hefei, 230012 China; 6 State Key Laboratory Breeding Base of Dao-di Herbs, National Resource Center for Chinese Materia Medica, China Academy of Chinese Medical Sciences, Beijing, China

**Keywords:** *Polygonatum cyrtonema* Hua, RNA-Seq, Transcriptome, Polysaccharides, Metabolic pathways, Gene expression

## Abstract

**Background:**

*Polygonatum cyrtonema* Hua (*P. cyrtonema*) is one of the most important herbs in traditional Chinese medicine. Polysaccharides in *P. cyrtonema* plants comprise a class of important secondary metabolites and exhibit a broad range of pharmacological functions.

**Results:**

In order to identify genes involved in polysaccharide biosynthesis, we performed RNA sequencing analysis of leaf, root, and rhizome tissues of *P. cyrtonema*. A total of 164,573 unigenes were obtained by assembling transcripts from all three tissues and 86,063 of these were annotated in public databases. Differentially expressed genes (DEGs) were determined based on expression profile analysis, and DEG levels in rhizome tissues were then compared with their counterparts in leaf and root tissues. This analysis revealed numerous genes that were either up-regulated or uniquely expressed in the rhizome. Multiple genes encoding important enzymes, such as UDP glycosyltransferases (UGTs), or transcription factors involved in polysaccharide biosynthesis were identified and further analyzed, while a few genes encoding key enzymes were experimentally validated using quantitative real-time PCR.

**Conclusion:**

Our results substantially expand the public transcriptome dataset of *P. cyrtonema* and provide valuable clues for the identification of candidate genes involved in metabolic pathways.

**Electronic supplementary material:**

The online version of this article (10.1186/s13007-019-0441-9) contains supplementary material, which is available to authorized users.

## Background

*Polygonatum cyrtonema* Hua (Liliaceae) is a medicinal and edible perennial plant that is used in traditional Chinese medicine for the treatment of coughs, dizziness, and lung problems [1]. According to the Chinese Pharmacopoeia, *P. cyrtonema* is often prescribed as the dried rhizome of *P. sibiricum* Red. Indeed, polysaccharides isolated from *P. cyrtonema* are the major bioactive component of herbal medicines and exhibit a range of important biological characteristics such as immunomodulatory, anti-aging, and antiviral properties [2, 3]. The content and composition of these polysaccharides in *P. cyrtonema* plants varies with geographic region, cultivation method, and individual age. This means that understanding the biosynthesis, metabolism, and regulation of polysaccharides in *P. cyrtonema* is of great significance.

Previously, high performance anion-exchange chromatography with pulsed amperometric detection (HPAEC-PAD) has been used to reveal that polysaccharides in *P. cyrtonema* include rhamnose, arabinose, galactose, glucose, mannose, and fructose [4]. The composition and structure of polysaccharides in *P. cyrtonema* show a remarkable level of diversity [5]; while polysaccharide biosynthesis in other medicinal plants remains poorly understood, studies on *Codonopsis pilosula* and *P. sibiricum* show that this process occurs via three main processes [6–8]. In the first place, sucrose is converted to glucose 1-phosphate (Glc-1P) and fructose 6-phosphate (Fru-6P). Secondly, β-fructofuranosidase (sacA) catalyzes the conversion of sucrose to glucose 6-phosphate (Glc-6P) as well as fructose [9] while hexokinase (HK) and fructokinase (scrk) convert fructose to Fru-6P [10, 11]. Phosphoglucomutase (pgm) is also involved in the isomerism of Glc-6P to Glc-1P [12] while UDP-glucose (UDP-Glc) is immediately formed from Glc-1P [13]. Subsequently, Fru-6P is indirectly converted to guanosine diphospho mannose (GDP-Man) [14]. A number of other NDP sugars are then generated from both UDP-Glc and GDP-Man by the action of nucleotide-diphospho-sugar (NDP-sugar) interconversion enzymes (NSEs) [15]. Plant polysaccharides are synthesized by numerous activated NDP-sugar precursors which are then added to polysaccharide and glycoconjugate residues via a series of glycosyltransferase (GT) reactions [16, 17].

A series of complete transcriptome and metabolomic functional analyses have been performed in the post-genomic era [18, 19]. In particular, RNA sequencing (RNA-Seq) has been shown to be the most efficient, cost effective method for the analysis of functional genes as well as for the accurate quantification of their expression in the absence of a reference genome [20–22]. Dozens of medicinal plants have so far been subjected to RNA-Seq analysis, including *Artemisia argyi* [23], *A. annua* [24], *Eugenia uniflora* L. [25], *P. sibiricum* [26], and *Dendrobium officinale* [27]. The use of this approach has enabled the identification of a number of new functional genes involved in specific metabolic pathways.

We conducted a comprehensive transcriptome analysis of *P. cyrtonema* plants and identified numerous genes involved in polysaccharide biosynthesis. The transcriptome data reported here are expected to provide as a foundation for future studies that address the molecular mechanisms of polysaccharide biosynthesis and will provide new insights on this species.

## Methods

### Plant material and RNA isolation

Whole *P. cyrtonema* plants were collected from the herb garden at the Anhui University of Chinese Medicine and identified by Professor Qingshan Yang (Anhui University of Chinese Medicine). Plant samples were cleaned with ultrapure water and dried on filter paper. The rhizomes, roots, and leaves of each were then separated and immediately frozen in liquid nitrogen (N). Separated tissues were selected from three replicates and pooled together and the total RNA of rhizomes, roots, and leaves was isolated using an RNA Plant Kit (Aidlab Biotech, Beijing, China) following the manufacturer’s instructions. RNA quality was evaluated using an Agilent 2100 BioAnalyzer (Agilent Technologies, Palo Alto, CA, USA) (Additional file 1: Table S1).

### Determination of total polysaccharide content

Polysaccharides were isolated from dried samples of *P. cyrtonema* rhizomes, roots, and leaves as described previously [28]. Briefly, dried powder (0.5 g) from each sample was mixed with 80% ethanol and was then extracted twice at 85 °C (1 h each time) in boiling distilled water. The precipitate was then collected and dissolved with distilled water. Solution absorbance was then determined using UV-spectrophotometry (JASCO Company, Japan). Anhydrous glucose was used as a standard; the standard curve of the relationship between concentration and absorbance is shown in Additional file 1: Fig. S1. The yield of total polysaccharides was calculated as follows:

$$\begin{aligned} Yield &= [{\text{Polysaccharide}}\;{\text{content}}\;{\text{extracted }}\,\left( {\text{g}} \right)\\ &\quad /{\text{Weight}}\;{\text{of}}\;{\text{dried}}\;{\text{powder}}\,\left( {\text{g}} \right)] \times 100\% \end{aligned}$$

### Construction of cDNA and RNA-Seq libraries

Total RNA was treated with DNase I to remove all traces of genomic DNA, and mRNA was purified using poly-T oligo-attached magnetic beads. Purified mRNA was then fragmented using divalent cations under elevated temperature in NEBNext First Strand Synthesis Reaction Buffer (5X). Samples of mRNA were then used for first-strand cDNA synthesis using reverse transcriptase and random hexamer primers, while second-strand cDNA was synthesized with RNase H and DNA Polymerase I using NEBNext double-stranded cDNA (ds cDNA) Fragmentase (New England BioLabs). Short cDNA fragments were recovered and repaired with NEBNext End Repair Module (New England BioLabs), and a single nucleotide (adenine) was added to 3ends using the NEBNext dA-Tailing Module (New England BioLabs). Fragments of cDNA were then ligated to the NEBNext Adaptor using the NEBNext Quick Ligation Module (New England BioLabs). In order to select appropriate cDNA fragments, a library was purified using the AMPure XP system (Beckman Coulter, Beverly, USA) while PCR amplification was performed using NEBNext Q5 Hot Start HiFi PCR Master Mix (2X), universal PCR primer, and index primer.

RNA-Seq libraries were generated using a NEBNext^®^ Ultra™ RNA Library Prep Kit for Illumina^®^ (NEB, USA), following the manufacturer’s instructions. The quality of each sample library was evaluated using an Agilent 2100 Bioanalyzer system (ABI, New York, NY, USA); qualified libraries were then sequenced and paired-end reads were generated using an Illumina HiSeq 4000 platform (Beijing Genomics Institute, Wuhan, China).

### RNA-Seq data analysis

Clean reads were obtained by discarding low-quality ones containing poly-N and adapter sequences using the SOAPnuke software [29] (parameters: -l 15 -q 0.2 -n 0.05 -i -A 0.25). Due to the absence of a reference genome, a transcriptome was assembled de novo from clean reads using Trinity (version 2.06) [30] applying the following parameters: –min_contig_length 150 –CPU 8 –min_kmer_cov 3 –min_glue 3 –bfly_opts ‘-V 5 –edge-thr = 0.1 –stderr’. Assembled transcripts were then clustered to remove redundancies using the Trinity and TGI clustering (TGICL) tool [31] (parameters: -l 40 -c 10 -v 25 -O ‘-repeat_stringency 0.95 -minmatch 35 -minscore 35’) and led to the identification of non-redundant sequences, termed unigenes.

### Functional annotation of unigenes

In order to perform functional annotation, unigenes were mapped to five databases using the BLAST (version 2.2.23, e-value $\leq$ 1e-5) software package released by the National Center of Biotechnology Information (NCBI) [32]. These five databases included those containing NCBI nucleotide (NT) sequences, NCBI non-redundant (NR) protein sequences, Clusters of Orthologous Groups of Proteins (COG), the Kyoto Encyclopedia of Genes and Genomes (KEGG), and a manually annotated and reviewed protein sequence database (SwissProt). Gene Ontology (GO) e-value = 1e−6) functional annotation was also performed using Blast2GO (version 2.5.0; default parameters) [33] with NR annotations, while InterPro annotations were constructed using InterProScan5 [34].

### Identification of differentially expressed genes (DEGs)

Clean transcriptome reads were mapped to unigenes using the software Bowtie2 (version 2.2.5) with default settings [35]. The read density of each sample was calculated using the fragments per kilobase of transcript per million (FPKM) method. Unigenes that exhibit differences in expression between two tissue types (i.e., rhizome vs. root and rhizome vs. leaf) at a fold change (FC) $\geq$ 2.00 and a false discovery rate (FDR) $\leq$ 0.001 were identified as DEGs using the PoissonDis method [36, 37]. These DEGs were then used for GO and KEGG enrichment analyses as previously described [38].

### Analysis of genes encoding transcription factors (TFs)

In order to determine the TF families represented in the *P. cyrtonema* transcriptome, the open reading frame (ORF) of each unigene was detected using the software getorf (EMBOSS:6.5.7.0) [39]. These ORFs were then aligned to all TF protein domains using the plant transcription factor database (PlnTFDB) via BLASTX (e-value $\leq$ 1e−5) and applying the hmmsearch method [40].

### Quantitative real-time PCR (qRT-PCR) analysis

In order to validate RNA-Seq data, qRT-PCR analysis was conducted using a QuantiNova SyBr Green PCR kit (Qiagen) on a PIKOREAL 96 Real-Time PCR System. Candidate reference qRT-PCR gene primers were designed using Primer Premier (version 5.0) (Additional file 1: Table S2). In this experiment, each qRT-PCR reaction contained 1 µl of diluted cDNA, 1 µl of each primer, 5 µl of 2X SYBR Green mix, and 2 µl of RNase-free water. All qRT-PCRs were performed using the following conditions: denaturation at 95 °C for 1 min, followed by 40 cycles of 95 °C for 20 s and then at 60 °C for 1 min. Successive qRT-PCR assays were performed using three biological and three technical replicates and to verify product specificity, melting curve analysis was performed after each amplification. The *actin* gene (CL16529.Contig1) of *P. cyrtonema* was used as a reference and the relative expression level of each unigene was calculated using the 2^−ΔΔCt^ approach [41].

## Results

### Total polysaccharide content of *P. cyrtonema* samples

We extracted polysaccharides from the dried rhizomes, roots, and leaves of *P. cyrtonema.* Results show that total polysaccharide content was highest in rhizomes (4.76%) and lowest in leaves (1.62%) (Additional file 1: Fig. S2).

### Illumina sequencing and de novo transcriptome assembly

A total of 10.42 Gb, 10.18 Gb, and 11.06 Gb of clean reads were obtained from 83.34 Mb, 81.63 Mb, and 89.13 Mb sets of raw data from *P. cyrtonema* leaf, root, and rhizome samples, respectively. All these data sets were characterized by Q30 $\geq$ 93.94%. These clean reads were then assembled sequentially so that full-length transcriptomes of each tissue could be reconstructed. A total of 164,573 unigenes were generated after selecting the longest transcript of each using the TGICL tool. These unigenes had a mean length of 710 bp and an N50 value of 1234 bp; 21.94% (36,108) and 39.84% (65,567) of these exceeded 1000 bp and 500 bp in length, respectively (Additional file 1: Fig. S3).

### Functional annotation and expression overview of unigenes

Out of the 164,573 unigenes identified in this analysis, 52.29% (86,063) were mapped to at least one public database; thus, 45.83%, 28.81%, 29.00%, 33.35%, 35.62%, 35.56%, and 13.76% unigenes were recorded as significant hits in the NR, NT, SwissProt, KEGG, KOG, InterPro, and GO databases, respectively (Table 1), and 20.22% unigenes (33,283) were co-annotated in all five databases (Additional file 1: Fig. S4A). Out of the 75,427 unigenes annotated in the NR database, 50.46%, 7.14%, 2.04%, and 35.68% were mapped to the genes of *Asparagus officinalis* (Liliaceae) [42], *Elaeis guineensis* (Arecaceae) [43], *Ananas comosus* (Bromeliaceae) [44], and others, respectively (Additional file 1: Fig. S4B). The GO enrichment analysis presented here divided these annotated unigenes into three classes: biological processes, cellular components, and molecular function. A total of 22,649 of these unigenes were then matched with one or more GO terms and comprise 54 functional groups (Additional file 1: Fig. S5). We found that ‘metabolic process’ and ‘biological regulation’ were the most abundant categories within biological processes, while within the molecular function term, ‘catalytic activity’ and ‘binding’ were the most abundant.

**Table 1:** Summary of *P. cyrtonema* unigenes annotated in seven public databases

Database	Number annotated	Annotated unigene ratio (%)
Nr	75,427	45.83
Nt	47,418	28.81
Swissprot	47,723	29
KEGG	54,881	33.35
KOG	58,627	35.62
Interpro	58,525	35.56
GO	22,649	13.76
Overall	86,063	52.29

**Fig. 1:**
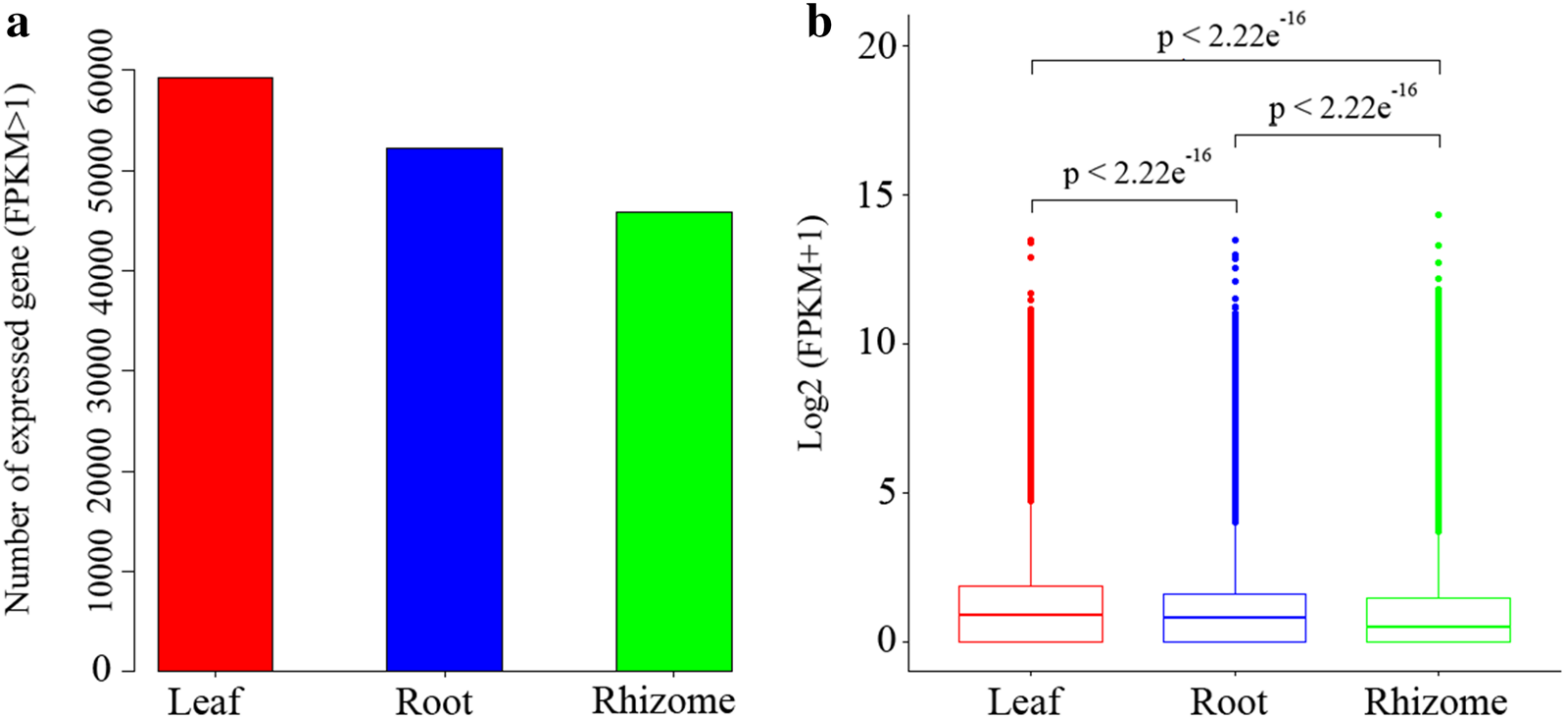
Expression profiles of genes in rhizome, leaf, and root tissues of *P. cyrtonema*. **a** Distributions of expressed unigenes (FPKM > 1) in the three tissues. **b** Boxplot of unigenes expressed in the three tissues. X-axis represents the tissues, and Y-axis shows the log_2_ (FPKM + 1) values. Significant test of the three tissues is performed using multi-independent sample Kruskal–Wallis test

Unigenes with FPKM > 1 were counted in each tissue. The results of this comparison showed that 59,271, 52,184, and 45,893 unigenes were expressed in leaves, roots, and rhizome samples, respectively (Fig. 1a). Gene expression level was highest in leaves compared with rhizomes and roots (Fig. 1b).

### Identification of genes involved in polysaccharide biosynthesis

**Fig. 2:**
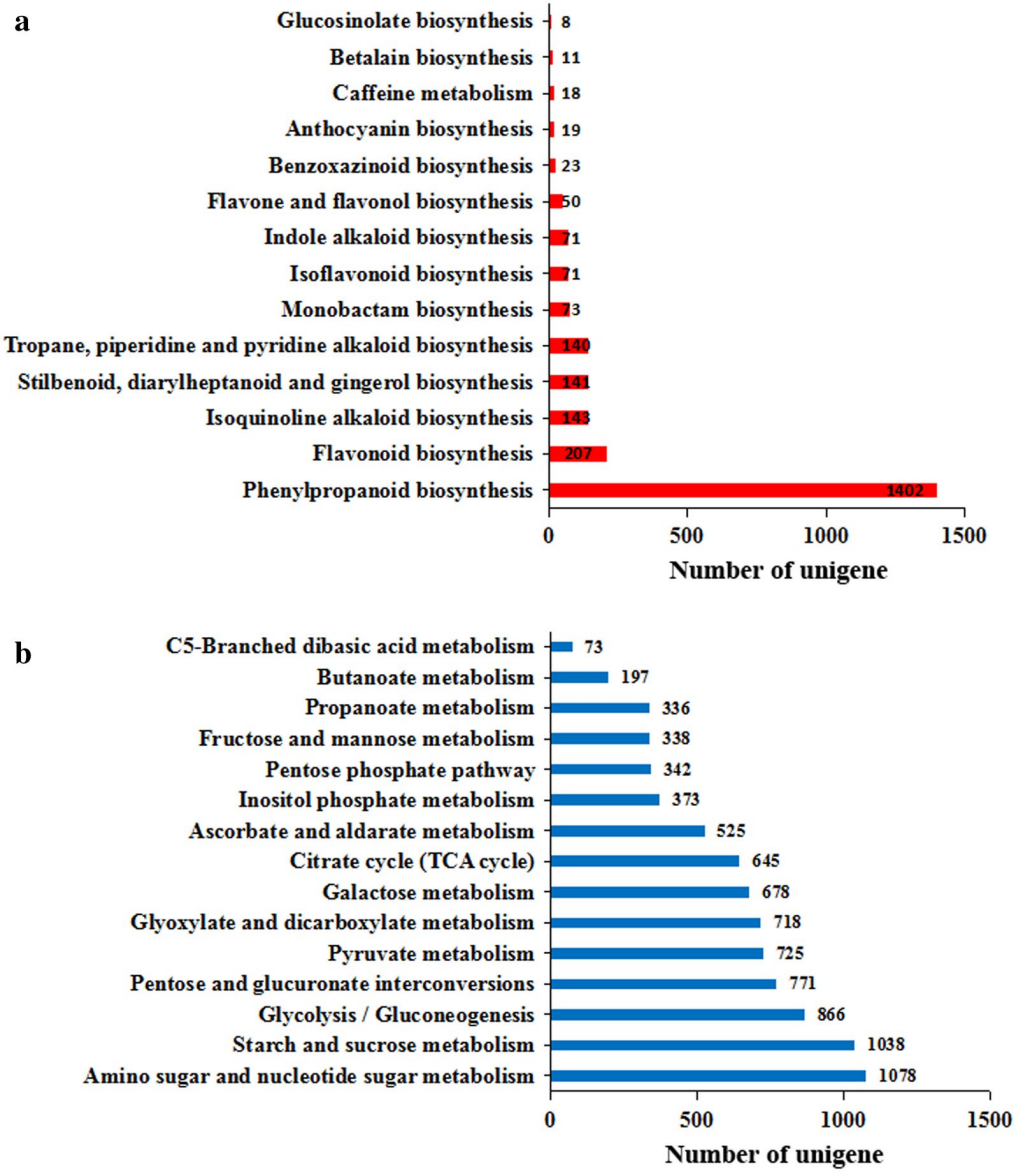
KEGG annotation of *P. cyrtonema* unigenes. **a** Pathway classifications for the biosynthesis of other secondary metabolites. **b** Pathway classifications for carbohydrate metabolism

In order to understand the most significant biological processes in *P. cyrtonema* plants, a total of 54,881 unigenes were annotated using the KEGG database and assigned to 136 pathways (19 subcategories) (Additional file 1: Fig. S6, Table S3). A total of 14 pathways were assigned to the biosynthesis of other secondary metabolites and the most abundant unigenes within this set were annotated within the phenylpropanoid biosynthesis pathway (Fig. 2a). The ‘carbohydrate metabolism’ subcategory included eight pathways with the largest number of unigenes (1078) involved in amino and nucleotide sugar metabolism. In addition, 3753 unigenes were matched with related polysaccharide biosynthesis pathways, including amino and nucleotide sugar metabolism, starch and sucrose metabolism, glycolysis/gluconeogenesis, and pentose and glucuronate interconversions (Fig. 2b).

**Fig. 3:**
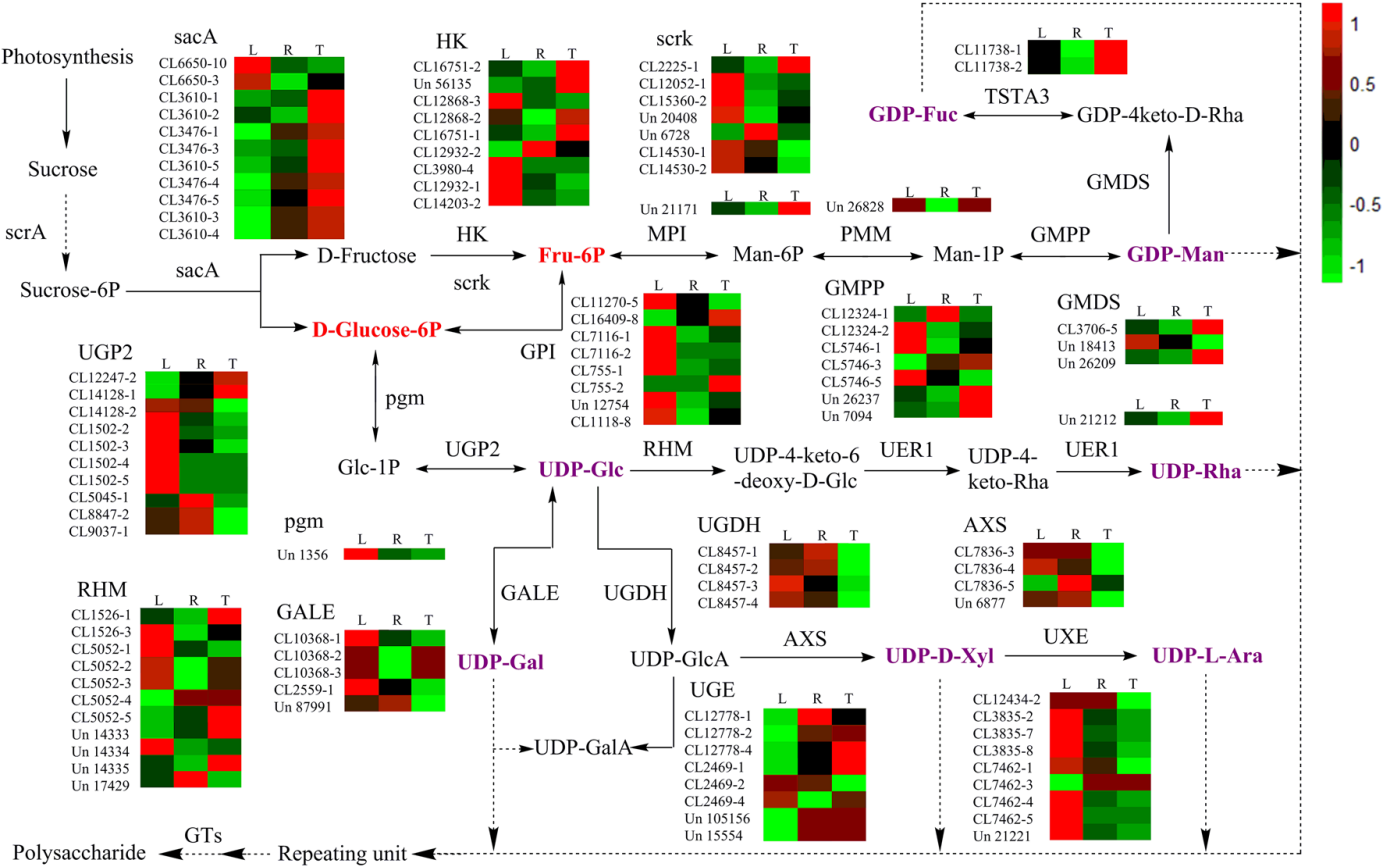
Proposed pathways for polysaccharide biosynthesis in *P. cyrtonema*. Activated monosaccharide units are marked in purple. Bold text in red background indicates key intermediates. Total expression levels of unigenes encoding enzymes involved in each step are shown. The columns L, R, and T represent leaf, root, and rhizome samples, respectively. Red and green represent high and low expression levels, respectively. Cluster of transcripts and Unigene are abbreviated as “CL” and “Un”, respectively. Arrows with solid lines represent the identified enzymatic reactions, and arrows with dashed lines represent multiple enzymatic reactions through multiple steps

In order to enhance our understanding of polysaccharide biosynthesis, we annotated 2116 unigenes involved in amino and nucleotide sugar metabolism (KO00520) as well as starch and sucrose metabolism (KO00500) pathways based on the KEGG database. A total of 337 unigenes encoding key enzymes, including UDP-glucose 4,6-dehydratase (RHM), 3,5-epimerase-4-reductase (UER1), GDP-mannose 4,6-dehydratase (GMDS), UDP-glucose 4-epimerase (GALE), UDP-glucose 6-dehydrogenase (UGDH), UDP-glucuronate 4-epimerase (UGE), and UDP-arabinose 4-epimerase (UXE) involved in the pathways discussed above were screened out (Table 2). We identified seven subclasses of NSEs within the *P. cyrtonema* transcriptome, specifically RHM (16 unigenes), UER1 (16 unigenes), GMDS (16 unigenes), GALE (13 unigenes), UGDH (7 unigenes), UGE (12 unigenes), and UXE (23 unigenes). These data enabled the identification of genes encoding enzymes involved in polysaccharide biosynthesis using the FPKM approach (Fig. 3).

**Table 2:** Number of unigenes encoding key enzymes involved in starch and sucrose biosynthesis and amino and nucleotide sugar metabolism in *P. cyrtonema*

Enzyme name	EC number	Unigene Number	No. in tubers	No. in roots	No. in leaves
β-Fructofuranosidase (sacA)	3.2.1.26	35	15	19	17
Hexokinase (HK)	2.7.1.1	24	8	11	8
Fructokinase (scrK)	2.7.1.4	14	6	5	7
Mannose-6-phosphate isomerase (MPI)	5.3.1.8	3	3	3	3
Phosphomannomutase (PMM)	5.4.2.8	10	4	6	7
Mannose-1-phosphate Guanylyltransferase (GMPP)	2.7.7.13	14	5	5	8
GDP-mannose 4,6-dehydratase (GMDS)	4.2.1.47	7	4	4	4
GDP-l-fucose synthase (TSTA3)	1.1.1.271	3	0	2	2
Glucose-6-phosphate isomerase (GPI)	5.3.1.9	17	9	10	9
Phosphoglucomutase (pgm)	5.4.2.2	11	5	6	5
UTP-glucose-1-phosphate Uridylyltransferase (UGP2)	2.7.7.9	117	52	64	64
UDP-glucose 4-epimerase (GALE)	5.1.3.2	13	7	8	8
UDP-glucuronate 4-epimerase (UGE)	5.1.3.6	12	4	4	4
UDP-glucose 6-dehydrogenase (UGDH)	1.1.1.22	7	4	2	2
UDP-apiose/xylose synthase (AXS)	AXS	7	4	5	2
UDP-arabinose 4-epimerase (UXE)	5.1.3.5	23	6	7	8
UDP-glucose 4,6-dehydratase (RHM)	4.2.1.76	16	6	10	9
3,5-Epimerase-4-reductase (UER1)	5.1.3.- 1.1.1.-	4	2	4	3

**Fig. 4:**
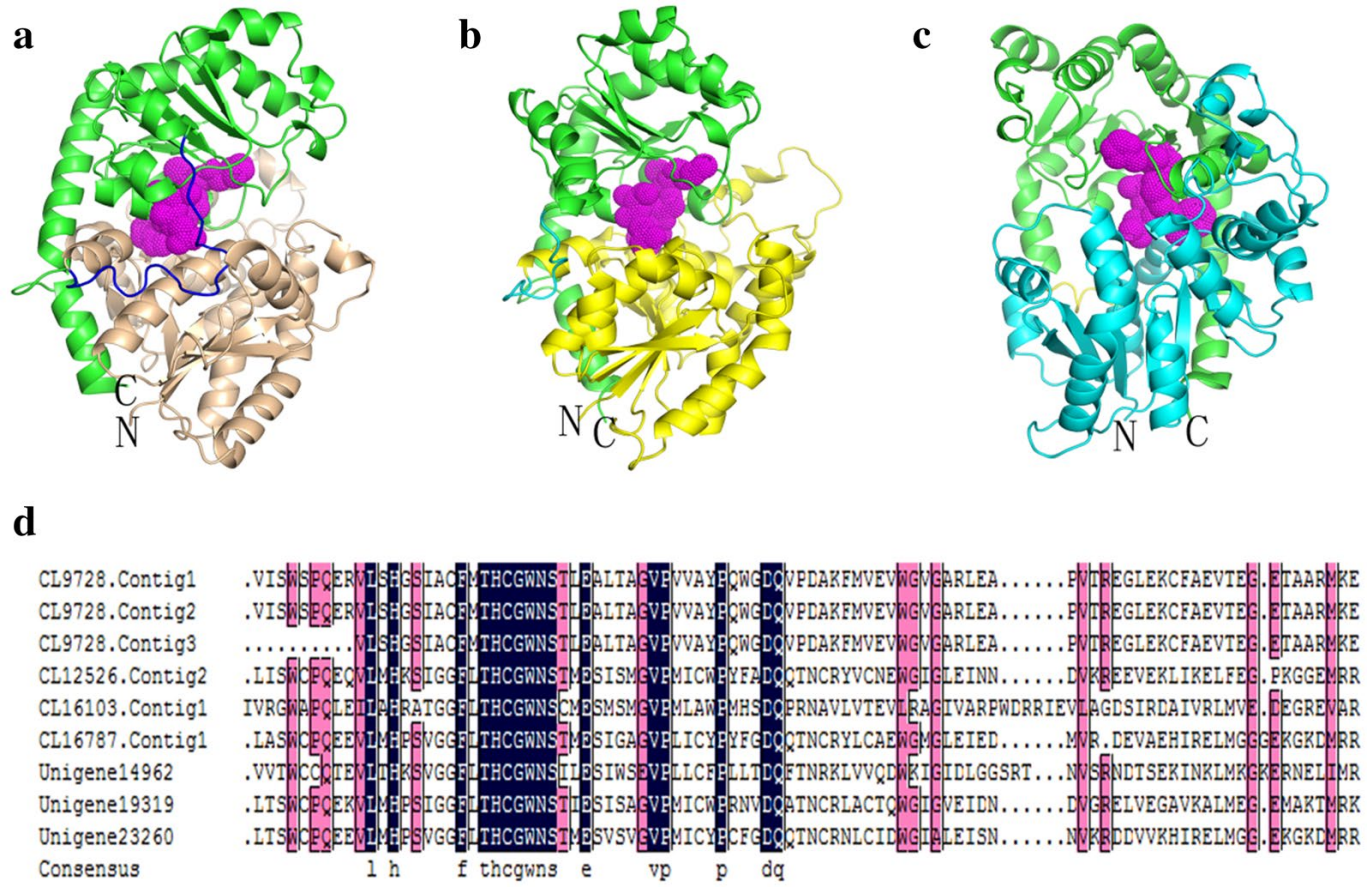
Three-dimensional (3D) model of UGTs and the signature motif of nine genes encoding UGTs in *P. cyrtonema*. **a**–**c** Model of UGTs CL12526.Contig2 (**a**), CL16787.Contig1 (**b**), Unigene23260 (**c**), and Template 2pq6.1.A, with sequence identity of 49.57%, 50.22%, and 51.81%, respectively. The C-terminal end (green) ranges from S282–L476, S255–K458, and G278–K501, respectively. The N-terminal end (wheat, yellow, and cyan, respectively) ranges from K9–Q263, K3–Q243, and K34–Q286, respectively. The highly conserved HCGWNS residues are shown as dots in magenta. **d** Amino acid sequence alignment of UGTs. Identical and similar amino acids are show in black and red, respectively. The alignment was performed using DNAMAN6.0.3.99 software

We also identified 27 unigenes encoding UDP glycosyltransferases (UGTs) (Additional file 1: Table S4). The amino acid sequence alignment of nine UGTs showed that a 29-amino acid region was well conserved within the C-terminal domain (Fig. 4d). We then constructed three-dimensional (3D) structural models of three UGTs (CL12526. Contig2, CL16787.Contig1, and Unigene23260) based on the 3D model of *Medicago truncatula* UGT85H2 (PDB ID: 2pq6) [45] using SWISS-MODEL (https://www.swissmodel.expasy.org/) and PyMOL. All UGT models comprised N- and C-terminal domains with similar Rossmann-type folds and contained either six or seven β-sheets flanked by eight or nine α-helices (Fig. 4a–c). This approach also showed that ‘HCGWNS’ residues were highly conserved (shown as magenta dots).

### Validation and expression analysis of genes encoding key enzymes

**Fig. 5:**
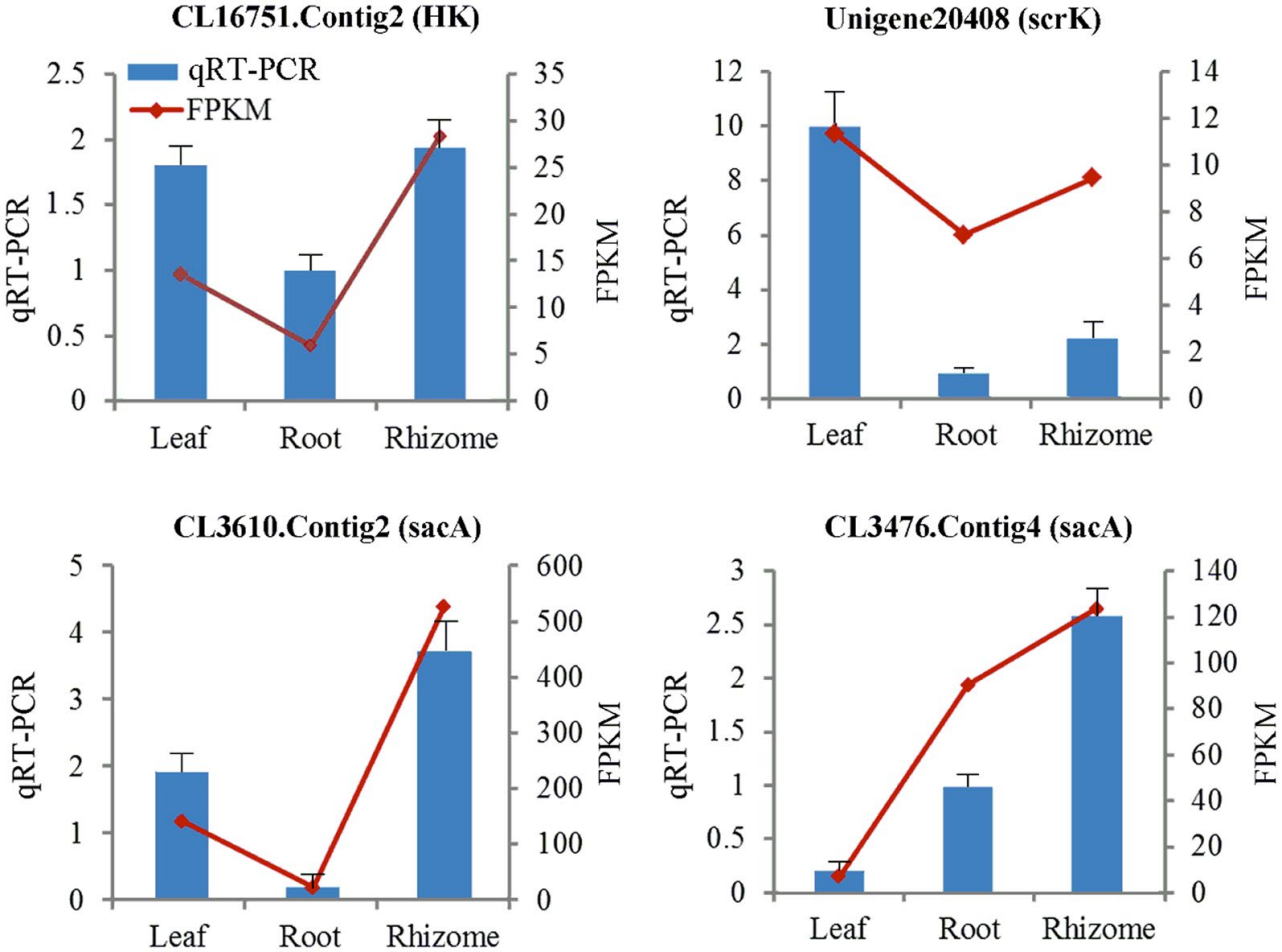
Expression analysis of four unigenes encoding enzymes involved in polysaccharide biosynthesis. Relative expression of CL16751.Contig2 (HK), Unigene20408 (scrk), and CL3476.Contig4 and CL3610.Contig2 (sacA) was analyzed by qRT-PCR. The *actin* gene (CL16529.Contig1) as a reference for normalization of three technical replicates. Root, rhizome, and leaf samples are used as normalizers in each experiment. Blue bars represent qRT-PCR data, and red lines indicate FPKM values. Data represent mean ± standard error of three replicates. The left Y-axis denotes relative expression levels of genes determined by qRT-PCR, and the right Y-axis denotes the FPKM values of RNA-Seq data

We tested the expression levels of genes encoding hexokinase (HK), fructokinase (scrk), and β-fructofuranosidase (sacA) using qRT-PCR assays (Fig. 5). Results revealed that the expression levels of genes encoding HK and sacA were highest in rhizomes, while those for genes encoding scrk were highest in leaves.

### Identification of DEGs

**Fig. 6:**
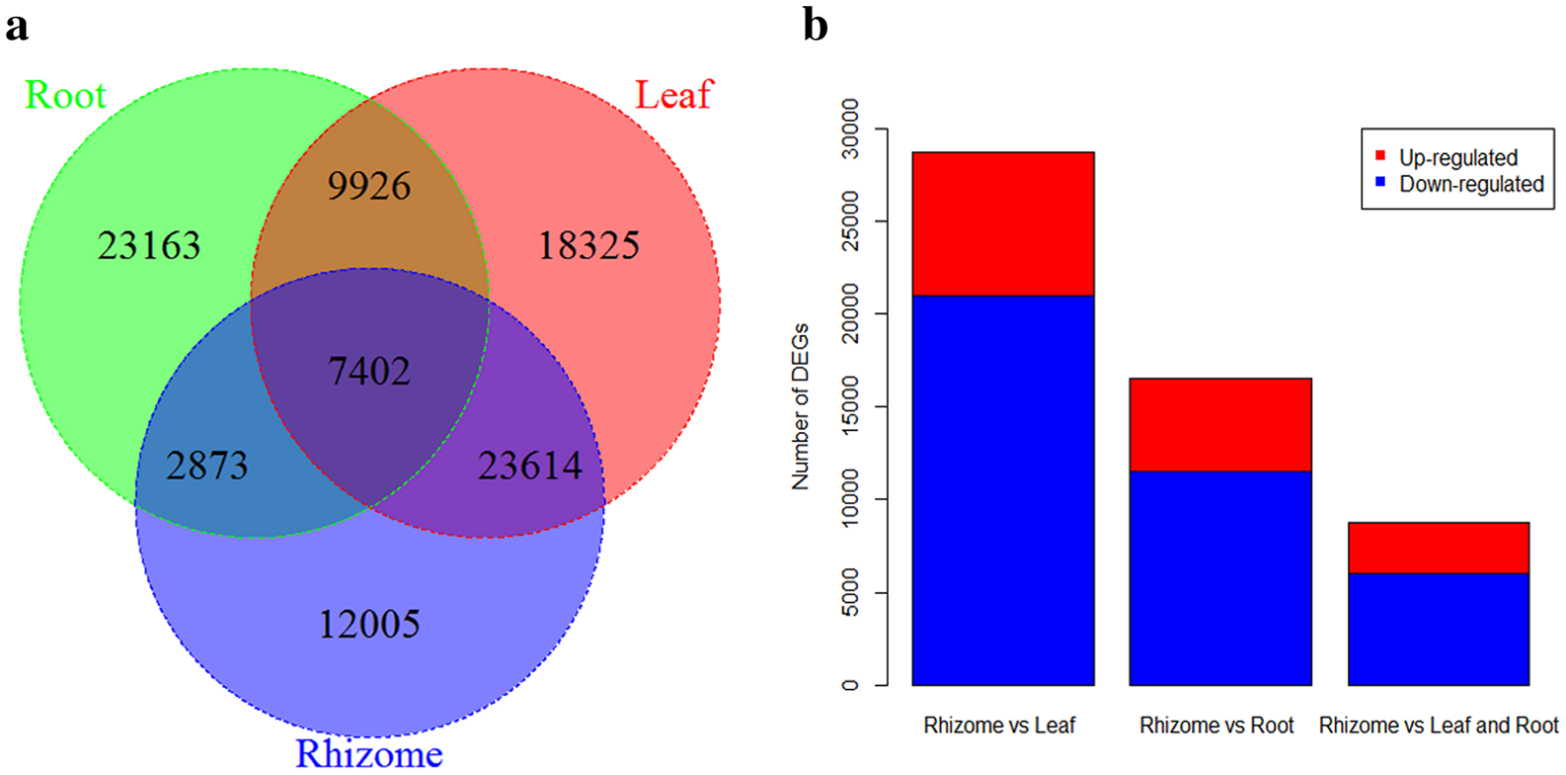
Unigenes expressed in rhizomes, leaves, and roots of *P. cyrtonema*. **a** Venn diagram of unigenes expressed in the three tissues. **b** Number of differentially expressed genes (DEGs) in the *P. cyrtonema* three tissues. The number of unigenes up-regulated or down-regulated in the specified samples are shown. DEGs with high or low expression levels in rhizomes compared with the other two types of tissues are defined as “up-regulated” and “down-regulated”, respectively

Amongst the unigenes identified in this study, 12,005 were expressed only in rhizomes, while 7402 were expressed in all three tissues (Fig. 6a). DEGs were identified in all three tissues using FPKM values for unigenes (Fig. 6b). A comparison of expression levels between rhizomes and leaves revealed 28,725 DEGs, of which 7739 were up-regulated and 20,986 were down-regulated in rhizomes compared with leaves. A comparison of expression levels between rhizomes and roots revealed 16,553 DEGs, of which 5016 were up-regulated and 11,537 were down-regulated in rhizomes compared with roots. A comparison of gene expression among all three tissues revealed 8774 DEGs, of which 2781 were up-regulated and 5993 were down-regulated in rhizomes compared with leaves and roots. The ‘carbohydrate metabolism’ subgroup was particularly enriched among DEGs; compared with leaves and roots, 772 and 475 unigenes were up-regulated in rhizomes, respectively (Table 3).

**Table 3:** Number of genes in the carbohydrate metabolic pathway up-regulated in the rhizome compared with leaf and root

Carbohydrate metabolism pathway	Pathway ID	Number of up-regulated genes	
		Rhizome versus leaf	Rhizome versus root
Amino sugar and nucleotide sugar metabolism	ko00520	106 (34.42%)	63 (27.27%)
Starch and sucrose metabolism	ko00500	123 (33.88%)	73 (39.67%)
Glycolysis/Gluconeogenesis	ko00010	70 (27.89%)	41 (26.28%)
Pentose and glucuronate interconversions	ko00040	96 (46.60%)	54 (34.39%)
Pyruvate metabolism	ko00620	50 (26.61%)	32 (25.00%)
Glyoxylate and dicarboxylate metabolism	ko00630	50 (27.03%)	29 (21.28%)
Galactose metabolism	ko00052	62 (32.12%)	46 (31.94%)
Citrate cycle (TCA cycle)	ko00020	36 (25.17%)	21 (21.00%)
Ascorbate and aldarate metabolism	ko00053	57 (38.26%)	29 (27.36%)
Inositol phosphate metabolism	ko00562	27 (23.68%)	12 (16.90%)
Pentose phosphate pathway	ko00030	39 (32.50%)	21 (33.33%)
Fructose and mannose metabolism	ko00051	32 (27.35%)	29 (35.80%)
Propanoate metabolism	ko00640	11 (12.50%)	14 (21.88%)
Butanoate metabolism	ko00650	9 (19.57%)	5 (15.63%)
C5-Branched dibasic acid metabolism	ko00660	4 (25.00%)	6 (42.86%)

### Analysis of genes showing rhizome-specific expression

A total of 2781 up-regulated DEGs showed rhizome-specific expression, with log_2_ FC > 1. We further explored these genes via GOSlim functional analysis; sequence homology analysis revealed that all 2781 DEGs mapped to at least one GO term while 932 mapped within biological processes, 1365 within cellular components, and 815 within molecular function (Additional file 1: Fig. S7). Specifically, within biological processes and molecular function categories, some genes were enriched under ‘metabolic process’, ‘biological regulation’, and ‘transcription regulator activity’ categories, indicating important metabolic activities within rhizomes.

**Fig. 7:**
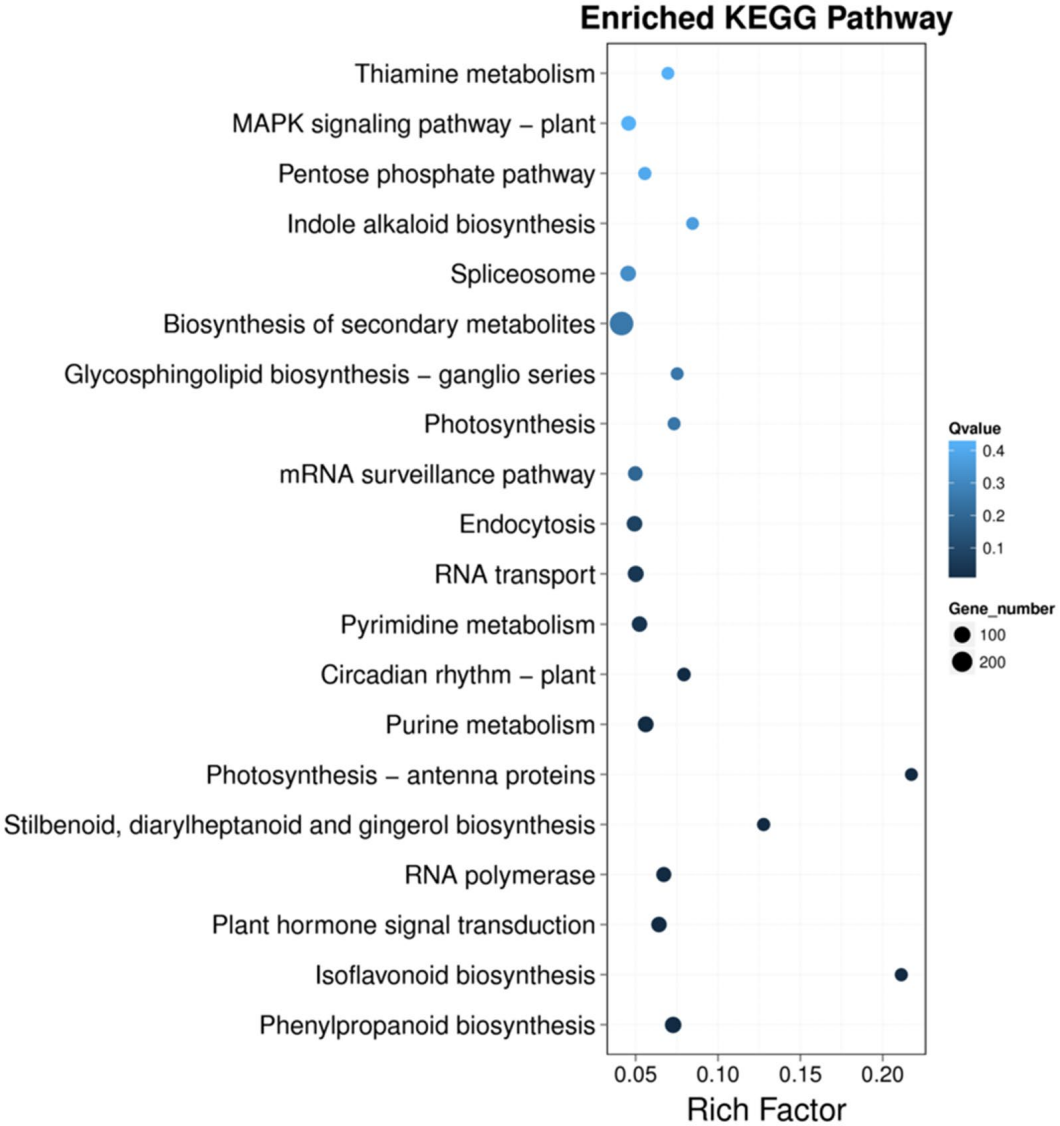
Rhizome-specific DEGs enriched in the KEGG pathway

All 2781 rhizome-specific DEGs were also annotated in the KEGG database. In order to further evaluate the biological functions of these DEGs, we compared them with the transcriptome of *P. cyrtonema*. Most DEGs were mapped to ‘translation’, ‘carbohydrate metabolism’, ‘transcription’, ‘biosynthesis of other secondary metabolites’, and ‘signal transduction’ (Additional file 1: Fig. S8). KEGG enrichment analysis revealed that these DEGs were significantly enriched in ‘biosynthesis of secondary metabolites’, ‘MAPK signaling pathway-plant’, ‘spliceosome’, ‘mRNA surveillance pathway’, ‘RNA transport’, ‘plant hormone signal transduction’, and ‘phenylpropanoid biosynthesis’ pathways (Fig. 7).

### Identification of TFs involved in the biosynthesis of polysaccharides and other secondary metabolites

**Fig. 8:**
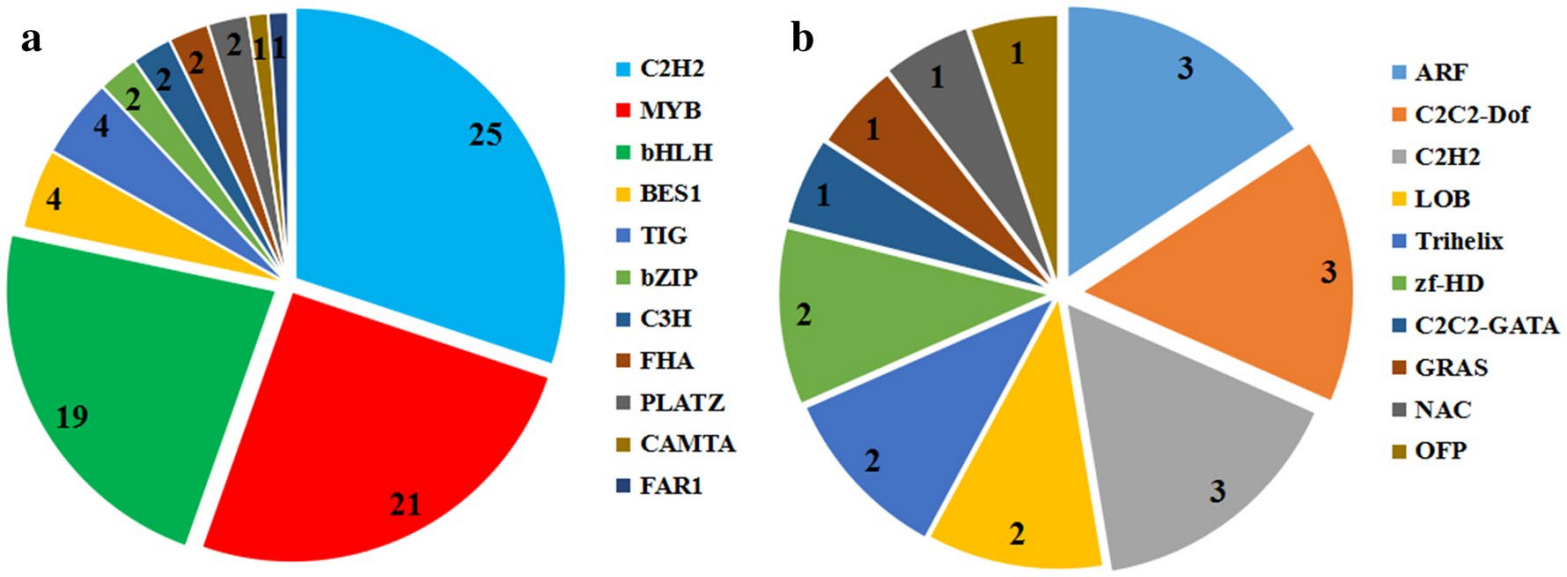
Transcription factors (TFs) involved in metabolic pathways. **a** TF families involved in carbohydrate metabolism. **b** TF families involved in the metabolism of other secondary metabolites

A total of 2531 putative unigenes encoding TFs were identified in the transcriptome database of *P. cyrtonema*, including 533 and 319 unigenes up-regulated in rhizomes compared with leaves and roots, respectively (Table 4). The major TF families identified in this analysis included MYB (314 unigenes), MYB-related (246 unigenes), AP2-EREBP (168 unigenes), WRKY (152 unigenes), bHLH (150 unigenes), C3H (124 unigenes), C2H2 (116 unigenes), and NAC (107 unigenes) groups. Results also showed that C2H2 (25 unigenes), MYB (21 unigenes), bHLH (19 unigenes), BES1 (four unigenes), TIG (four unigenes), bZIP, C3H, FHA, and PLATZ (two unigenes each), and CAMTA and FAR1 (one unigene each) TFs participated in carbohydrate metabolism. Results show that a total of 19 TF families participated in the biosynthesis of other secondary metabolites (Fig. 8a, b).

**Table 4:** Type and number of transcription factors (TFs) encoded by the DEGs of *P. cyrtonema*

TF_family	Number of unigenes	Up-regulated unigenes in rhizome versus leaf	Up-regulated unigenes in rhizome versus root
MYB	314	72	26
MYB-related	246	51	14
AP2-EREBP	168	34	17
WRKY	152	61	35
bHLH	150	38	22
C3H	124	21	6
C2H2	116	26	35
NAC	107	30	15
FAR1	93	9	7
ABI3VP1	72	11	4
GRAS	67	11	15
Zn-clus	62	0	0
G2-like	60	5	8
mTERF	59	20	0
C2C2-GATA	45	11	6
FHA	45	2	1
Trihelix	41	12	19
HSF	40	6	11
ARF	40	14	7
MADS	36	5	6
Tify	36	5	5
bZIP	32	4	2
TCP	30	14	5
C2C2-Dof	28	2	2
SBP	28	2	1
TUB	27	5	1
Other	313	62	49
Total number	2531	533	319

## Discussion

The herb *P. cyrtonema* is very commonly used in traditional Chinese medicine for the treatment of various human conditions because of its immunomodulatory, anti-aging, and antiviral activities. Although polysaccharides are the major active constituents of *P. cyrtonema* plants, genomic data remain unavailable in spite of the significance of this species in treating various health conditions. Little is also known about the mechanisms underlying polysaccharide biosynthesis and metabolism in *P. cyrtonema*. The aim of this study was therefore to identify genes involved in polysaccharide biosynthesis and metabolism. This information will facilitate the further investigation of *P. cyrtonema* and the identification of key markers of polysaccharide biosynthesis and metabolism.

Three different types of tissues were utilized in this study for RNA-Seq analysis using Illumina HiSeq 4000. Transcriptome analysis of *P. cyrtonema* using RNA-Seq generated 31.66 Gb of clean reads that were assembled into 164,573 unigenes with an average length of 710 bp. This approach therefore enabled an understanding of polysaccharide biosynthesis at the molecular level. A total of 86,063 unigenes were identified using BLAST, of which 47.71% remained unannotated in the NCBI database because of insufficient information available in the publicly available plant transcriptome and genomic databases. Comparisons with the transcriptomes of other medicinal plants showed that *P. cyrtonema* harbors a higher number of unigenes than *P. sibiricum* (74,130) [26]. The unigenes of *P. cyrtonema* exhibited a homogeneous size distribution and 24.43% of these unigenes (40,206) were longer than 1000 bp. Our transcriptome results are of higher quality and reliability than those previously assembled.

The best hit for each unigene was then queried against the NCBI NR database and a functional GO annotation was assigned in terms of cellular components, biological processes, and molecular function. A large number of diverse GO terms were assigned to these unigenes, indicating remarkable diversity within the transcriptome of *P. cyrtonema* leaf, root, and rhizome. Numerous unigenes involved in polysaccharide biosynthesis were identified via KEGG annotations and the total expression levels of genes encoding various enzymes were determined based on FPKM values (Fig. 3).

The expression levels of unigenes encoding sacA, RHM, MPI, GMDS, TSTA3, UER1, and UGE enzymes were higher in rhizomes compared with leaves and roots. In contrast, total unigene expression levels encoding HK, scrk, UGP2, GPI, GMPP, GALE, UXE, and UGDH were higher in leaves. Previous studies have shown that HK, scrk, and sacA play important roles in regulating carbohydrate metabolism as the expression levels of genes encoding these enzymes influence its accumulation [46–50]. The expression levels of genes encoding these enzymes were also examined using qRT-PCR which confirmed the reliability of our transcriptome data (Fig. 5). Higher expression levels of genes encoding HK (CL16751.Contig2) and sacA (CL3476.Contig4 and CL3610.Contig2) in rhizomes (as analyzed by qRT-PCR) are consistent with higher polysaccharide accumulation in *P. cyrtonema* rhizomes, as revealed by UV spectrophotometry (Additional file 1: Fig. S2). This result indicates that these genes play critical roles in regulating the polysaccharide content of *P. cyrtonema* rhizomes. The characterization of these unigenes will be useful for understanding the molecular mechanisms underlying polysaccharide biosynthesis. The expression levels recorded here for the gene encoding scrk (Unigene 20,408) in rhizomes versus leaves were inconsistent with those for the other genes involved in polysaccharide biosynthesis. These data are consistent with RNA-Seq analysis of *P. cyrtonema* and confirm the reliability of our RNA-Seq approach. The gene encoding scrk possesses numerous transcripts and has a synergistic effect on the regulation of polysaccharide biosynthesis. The qRT-PCR data on scrk transcripts obtained in this study may not be positively correlated with polysaccharide biosynthesis in the rhizome and several methodological challenges associated with RNA-seq analysis remain, including fragmentation, length, and transcriptome composition biases [51, 52]. These factors all lead to biased gene expression results.

It is clear that UGTs belong to a super family of enzymes and catalyze the addition of a glycosyl group from a nucleotide sugar (UDP glucuronic acid, UDP galactose, UDP glucose, or UDP xylose) to a small hydrophobic molecule [53]. A total of 27 unigenes encoding UGTs were identified here within the *P. cyrtonema* transcriptome. Amino acid sequence alignments for nine of the 27 UGTs with complete ORFs revealed a signature motif, specifically the plant secondary product glycosyltransferase one [54] which contains 29 conserved amino acids (Fig. 4d). This region is consistent with the consensus signature sequence seen in other plants and mammalian UGTs [55]: (FVA)-(LIVMF)-(TS)-(HQ)-(SGAC)-G-X(2)-(STG)-X(2)-(DE)-X(6)-P-(LIVMFA)-(LIVMFA)-X(2)-P-(LMVFIQ)-X(2)-(DE)-Q. In this sequence, conserved amino acids are listed within parentheses and X denotes any amino acid. This motif has long been suspected to be the binding site for nucleotide-activated sugars, based on comparisons with crystallographic data from bacterial enzymes. A UGT model (UGT73A5) from *Dorotheanthus bellidiformis* (Aizoaceae) indicates direct interaction with the highly conserved ‘HCGWNS’ residues and the uracil moiety of UDP-glucose [56] with the last amino acid of the plant secondary product glycosyltransferase motif ‘Q’; this result can be confirmed via discrimination between UDP-glucose and UDP-galactose [57]. Studies investigating the relationship between the structure and activity of UGTs suggest that the N-terminal domain acts as the acceptor binding site, while the C-terminal domain acts as the donor response site [54]. The 3D structural models of all three UGTs showed N- and C-terminal domains with similar Rossmann-type folds linked together via a long loop (Fig. 4a–c). Although the three genes encoding UGTs exhibit sequence diversity, their protein spatial structures are nevertheless conservative and suggest similar functions.

Results show that TFs are associated with a variety of plant biological processes. A large number of these have been isolated and shown to play major roles in the biosynthesis of polysaccharides and other secondary metabolism processes. A total of 1131 candidate TFs were assigned to the MYB, AP2-EREBP, WRKY, bHLH, C3H, C2H2, and NAC families; these TFs likely play roles in regulating polysaccharide biosynthesis. Over-expression of the gene encoding MYB46 in *Arabidopsis thaliana* resulted in a significant increase in the mannan content of hemicellulosic polysaccharides [58]. A total of 314 candidate unigenes encoding MYB TFs were identified here, of which 72 and 26 were up-regulated in the rhizome compared with leaves and roots, respectively (Table 4). These up-regulated unigenes are important candidates for future analyses aimed at investigating the regulation of polysaccharide biosynthesis in *P. cyrtonema*. At the same time, RNA-seq cannot be used to sufficiently detect genes or transcripts with low expression levels and are especially important for regulatory genes [59, 60]. The genes doublesex (dsx) and fruitless (fru), transcription factors (TFs) involved in sexual dimorphism in flies, cannot be determined by RNA-Seq in the deeply sequenced modENCODE embryo samples with low expression [61]. These genes will influence expression estimates for transcripts as well as their functional research.

## Conclusion

We conducted a comprehensive RNA-Seq analysis of leaves, roots, and rhizome tissues of *P. cyrtonema* plants and were able to identify numerous genes involved in polysaccharide biosynthesis. The results of RNA-Seq analysis were validated using qRT-PCR for a few genes. Our results help explain the accumulation of secondary metabolites. The transcriptome data reported here will facilitate future studies on the molecular mechanisms of polysaccharide biosynthesis and provide new insights into *P. cyrtonema*.

## Additional file

**Additional file 1: Table S1.** RNA information of different tissues. **Fig. S1.** Standard curve of glucose at 582 nm. **Table S2.** Gene descriptions and primers used for qRT-PCR. **Fig. S2.** Total polysaccharides content from rhizomes, roots and leaves of P. cyrtonema. **Fig. S3.** The length distribution of unigenes for P. cyrtonema transcriptome assembly. **Fig. S4A and Fig. S4B.** (A) Venn diagram of annotated unigenes from the different databases. (B) Species distribution annotated in the NR database for P. cyrtonema. **Fig. S5.** GO function annotation of P. cyrtonema transcriptome. **Fig. S6, Table S3.** KEGG functional classifications of the annotated unigenes in P. cyrtonema. KEGG annotation of all unigenes. **Table S4.** UDP glycosyltransferases annotated in P. cyrtonema. **Fig. S7.** GOSlim analysis of rhizome-specifc up-regulation genes. **Fig. S8.** KEGG functional classifications of the rhizome-specifc up-regulation genes in P. cyrtonema.

## References

[1] Luo M, Zhang WW, Deng CF, Tan QS, Luo C, Luo S (2016). Advances in studies of medicinal crop *Polygonatum cyrtonema* Hua. Lishizhen Med Mater Med Res.

[2] Gu H, Meng Y, Pu Q (2003). Polysaccharide from *Polygonatum cyrtonema* Hua against herpes simplex virus in vitro. Chin J Appl Environ Biol.

[3] Liu XX, Wan ZJ, Shi L, Lu XX (2011). Preparation and antiherpetic activities of chemically modified polysaccharides from *Polygonatum cyrtonema* Hua. Carbohydr Polym.

[4] Lian-Jun HE, Gan YP, Wei-De LV, Rao JF, Yang JM, Jia-Sheng YU (2017). Monosaccharide composition analysis on polysaccharides in *Polygonatum cyrtonema* by high performance anion-exchange chromatography with pulsed amperometric detection. Chin Tradit Herb Drugs.

[5] Liu F, Liu Y, Meng Y, Yang M, He K (2004). Structure of polysaccharide from *Polygonatum cyrtonema* Hua and the antiherpetic activity of its hydrolyzed fragments. Antivir Res.

[6] Peng J, Zhao S, Wu X, Liu D, Hu Z, Xu Z (2000). cDNA cloning and structural analysis of granule-bound starch synthase gene of hairy roots of Astragalus membranaceus. Acta Bot Sin.

[7] Jian PG, Dong W, Ling YC, Hai FS (2015). Transcriptome sequencing of *Codonopsis pilosula* and identification of candidate genes involved in polysaccharide biosynthesis. PLoS ONE.

[8] Wang S, Wang B, Hua W, Niu J, Dang K, Qiang Y, Wang Z (2017). De novo assembly and analysis of *Polygonatum sibiricum* transcriptome and identification of genes involved in polysaccharide biosynthesis. Int J Mol Sci.

[9] Xie Y, Zhou H, Liu C, Jing Z, Ning L, Zhao Z, Sun G, Zhong Y (2017). A molasses habitat-derived fungus *Aspergillus tubingensis* XG21 with high β-fructofuranosidase activity and its potential use for fructooligosaccharides production. AMB Express.

[10] Zhang Y, Zhen L, Tan X, Li L, Wang X (2014). The involvement of hexokinase in the coordinated regulation of glucose and gibberellin on cell wall invertase and sucrose synthesis in grape berry. Mol Biol Rep.

[11] Perezcenci M, Salerno GL (2014). Functional characterization of *Synechococcus amylosucrase* and fructokinase encoding genes discovers two novel actors on the stage of cyanobacterial sucrose metabolism. Plant Sci.

[12] Uematsu K, Suzuki N, Iwamae T, Inui M, Yukawa H (2012). Expression of Arabidopsis plastidial phosphoglucomutase in tobacco stimulates photosynthetic carbon flow into starch synthesis. J Plant Physiol.

[13] Park JI, Ishimizu T, Suwabe K, Sudo K, Masuko H, Hakozaki H, Nou IS, Suzuki G, Watanabe M (2010). UDP-glucose pyrophosphorylase is rate limiting in vegetative and reproductive phases in *Arabidopsis thaliana*. Plant Cell Physiol.

[14] Bachmann P, Zetsche K (1979). A close temporal and spatial correlation between cell growth, cell wall synthesis and the activity of enzymes of mannan synthesis in *Acetabularia mediterranea*. Planta.

[15] Yin Y, Huang J, Gu X, Barpeled M, Xu Y (2011). Evolution of plant nucleotide-sugar interconversion enzymes. PLoS ONE.

[16] Breton C, Snajdrová L, Jeanneau C, Koca J, Imberty A (2006). Structures and mechanism of glycosyltransferases. Glycobiology.

[17] Pauly M, Gille S, Liu L, Mansoori N, de Souza A, Schultink A, Xiong G (2013). Hemicellulose biosynthesis. Planta.

[18] Matsuda F, Hirai MY, Sasaki E, Akiyama K, Yonekura-Sakakibara K, Provart NJ, Sakurai T, Shimada Y, Saito K (2010). AtMetExpress development: a phytochemical atlas of Arabidopsis development. Plant Physiol.

[19] Saito K, Hirai MY, Yonekura-Sakakibara K (2008). Decoding genes with coexpression networks and metabolomics—‘majority report by precogs’. Trends Plant Sci.

[20] Metzker ML (2010). Sequencing technologies—the next generation. Nat Rev Genet.

[21] Tang F, Barbacioru C, Wang Y, Nordman E, Lee C, Xu N, Wang X, Bodeau J, Tuch BB, Siddiqui A (2009). mRNA-Seq whole-transcriptome analysis of a single cell. Nat Methods.

[22] Zhang H, He L, Cai L (2018). Transcriptome sequencing: RNA-Seq. Computational systems biology.

[23] Liu M, Zhu J, Wu S, Wang C, Guo X, Wu J, Zhou M (2018). De novo assembly and analysis of the Artemisia argyi transcriptome and identification of genes involved in terpenoid biosynthesis. Sci Rep.

[24] Wei W, Wang Y, Zhang Q, Yan Q, Guo D (2009). Global Characterization of Artemisia annua glandular trichome transcriptome using 454 pyrosequencing. BMC Genom.

[25] Guzman F, Kulcheski FR, Turchettozolet AC, Margis R (2014). De novo assembly of *Eugenia uniflora* L. transcriptome and identification of genes from the terpenoid biosynthesis pathway. Plant Sci.

[26] Wang S, Wang B, Hua W, Niu J, Dang K, Qiang Y, Wang Z (2017). De novo assembly and analysis of *Polygonatum sibiricum* transcriptome and identification of genes involved in polysaccharide biosynthesis. Int J Mol Sci.

[27] He C, Zhang J, Liu X, Zeng S, Wu K, Yu Z, Wang X, Ja TDS, Lin Z, Duan J (2015). Identification of genes involved in biosynthesis of mannan polysaccharides in *Dendrobium officinale* by RNA-seq analysis. Plant Mol Biol.

[28] Li F, Gao J, Xue F, Yu X, Shao T (2016). Extraction Optimization, Purification and Physicochemical Properties of Polysaccharides from Gynura medica. Molecules.

[29] Chen Y, Chen Y, Shi C, Huang Z, Zhang Y, Li S, Li Y, Ye J, Yu C, Li Z (2018). SOAPnuke: a MapReduce acceleration supported software for integrated quality control and preprocessing of high-throughput sequencing data. Gigascience.

[30] Grabherr MG, Haas BJ, Yassour M, Levin JZ, Thompson DA, Amit I, Adiconis X, Fan L, Raychowdhury R, Zeng Q (2011). Full-length transcriptome assembly from RNA-Seq data without a reference genome. Nat Biotechnol.

[31] Pertea G, Huang X, Liang F, Antonescu V, Sultana R, Karamycheva S, Lee Y, White J, Cheung F, Parvizi B (2003). TIGR Gene Indices clustering tools (TGICL): a software system for fast clustering of large EST datasets. Bioinformatics.

[32] Wm W, Myers EW, Lipman DJ (2008). : Blast (basic local alignment search tool). Encycl Genet Genom Proteomics Inform.

[33] Conesa A, Götz S, Garcíagómez JM, Terol J, Talón M, Robles M (2005). Blast2GO: a universal tool for annotation, visualization and analysis in functional genomics research. Bioinformatics.

[34] Quevillon E, Silventoinen V, Pillai S, Harte N, Mulder N, Apweiler R, Lopez R (2005). InterProScan: protein domains identifier. Nucl Acids Res.

[35] Langmead B, Salzberg SL (2012). Fast gapped-read alignment with Bowtie 2. Nat Methods.

[36] Chen Z, Liu J, Ng HKT, Nadarajah S, Kaufman HL, Yang JY, Deng Y (2011). Statistical methods on detecting differentially expressed genes for RNA-seq data. BMC Syst Biol.

[37] Stegle O, Drewe P, Bohnert R, Borgwardt K, Rätsch G (2010). Statistical tests for detecting differential RNA-transcript expression from read counts. Nat Preced.

[38] Audic S, Claverie JM (1997). The significance of digital gene expression profiles. Genome Res.

[39] Rice P, Longden I, Bleasby A (2000). EMBOSS: the European molecular biology open software suite. Trends Genet.

[40] Mistry J, Finn RD, Eddy SR, Bateman A, Punta M (2013). Challenges in homology search: HMMER3 and convergent evolution of coiled-coil regions. Nucl Acids Res.

[41] Livak KJ, Schmittgen TD (2001). Analysis of relative gene expression data using real-time quantitative PCR and the 2(-Delta Delta C(T)) Method. Methods.

[42] Li X-M, Cai J-L, Wang W-X, Ai H-L, Mao Z-C (2015). Two new acetylenic compounds fromAsparagus officinalis. J Asian Nat Prod Res.

[43] Xiao Y, Zhou L, Lei X, Cao H, Wang Y, Dou Y, Tang W, Xia W (2017). Genome-wide identification of WRKY genes and their expression profiles under different abiotic stresses in *Elaeis guineensis*. PLoS ONE.

[44] Lin YS, Kuan CS, Weng IS, Tsai CC (2015). Cultivar identification and genetic relationship of pineapple (*Ananas comosus*) cultivars using SSR markers. Genet Mol Res GMR.

[45] Li L, Modolo LV, Escamilla-Trevino LL, Achnine L, Dixon RA, Wang X (2007). Crystal structure of Medicago truncatula UGT85H2–insights into the structural basis of a multifunctional (iso)flavonoid glycosyltransferase. J Mol Biol.

[46] Abhijit K, Xiaoxia X, Moore BD (2012). Arabidopsis Hexokinase-Like1 and Hexokinase1 form a critical node in mediating plant glucose and ethylene responses. Plant Physiol.

[47] Kanayama Y, Granot D, Dai N, Petreikov M, Schaffer A, Powell A, Bennett AB (1998). Tomato fructokinases exhibit differential expression and substrate regulation. Plant Physiol.

[48] Stein O, Granot D (2018). Plant Fructokinases: evolutionary, developmental, and metabolic aspects in sink tissues. Front Plant Sci.

[49] Xie Y, Zhou H, Liu C, Zhang J, Li N, Zhao Z, Sun G, Zhong Y (2017). A molasses habitat-derived fungus *Aspergillus tubingensis* XG21 with high β-fructofuranosidase activity and its potential use for fructooligosaccharides production. Amb Express.

[50] Zhang J, Liu C, Xie Y, Li N, Ning Z, Du N, Huang X, Zhong Y (2017). Enhancing fructooligosaccharides production by genetic improvement of the industrial fungus Aspergillus niger ATCC 20611. J Biotechnol.

[51] Wang Z, Gerstein M, Snyder M (2009). RNA-Seq: a revolutionary tool for transcriptomics. Nat Rev Genet.

[52] Finotello F, Di Camillo B (2015). Measuring differential gene expression with RNA-seq: challenges and strategies for data analysis. Brief Funct Genom.

[53] Mackenzie PI, Owens IS, Burchell B, Bock KW, Bairoch A, Bélanger A, Fournel-Gigleux S, Green M, Hum DW, Iyanagi T (1997). The UDP glycosyltransferase gene superfamily: recommended nomenclature update based on evolutionary divergence. Pharmacogenet Genomics.

[54] Gachon CMM, Langlois-Meurinne M, Saindrenan P (2005). Plant secondary metabolism glycosyltransferases: the emerging functional analysis. Trends Plant Sci.

[55] Meech R, Mackenzie PI (2010). Structure and function of uridine diphosphate glucuronosyltransferases. Clin Exp Pharmacol Physiol.

[56] Judith H, Wolfgang B, Thomas V (2010). Site-directed mutagenesis and protein 3D-homology modelling suggest a catalytic mechanism for UDP-glucose-dependent betanidin 5-o-glucosyltransferase from Dorotheanthus bellidiformis. Plant J Cell Mol Biol.

[57] Kubo A, Arai Y, Nagashima S, Yoshikawa T (2004). Alteration of sugar donor specificities of plant glycosyltransferases by a single point mutation. Arch Biochem Biophys.

[58] Kim WC, Reca IB, Kim YS, Park S, Thomashow MF, Keegstra K, Han KH (2014). Transcription factors that directly regulate the expression of CSLA9 encoding mannan synthase in Arabidopsis thaliana. Plant Mol Biol.

[59] Jiang Z, Zhou X, Li R, Michal JJ, Zhang S, Dodson MV, Zhang Z, Harland RM (2015). Whole transcriptome analysis with sequencing: methods, challenges and potential solutions. Cellular and molecular life sciences: CMLS.

[60] Malone JH, Oliver B (2011). Microarrays, deep sequencing and the true measure of the transcriptome. BMC Biol.

[61] Graveley BR, Brooks AN, Carlson JW, Duff MO, Landolin JM, Yang L, Artieri CG, van Baren MJ, Boley N, Booth BW (2011). The developmental transcriptome of Drosophila melanogaster. Nature.

