# Supplementary material for: Transcriptome analysis of Polygonatum cyrtonema Hua: identification of genes involved in polysaccharide biosynthesis
Source: Plant Methods. 2019 Jun 26;15:65. doi: 10.1186/s13007-019-0441-9 (PMC6593569; doi:10.1186/s13007-019-0441-9)
Supplement: Supplementary file 1 — Additional file 1: Table S1. RNA information of different tissues. Fig. S1. Standard curve of glucose at 582 nm. Table S2. Gene descriptions and primers used for qRT-PCR. Fig. S2. Total polysaccharides content from rhizomes, roots and leaves of P. cyrtonema. Fig. S3. The length distribution of unigenes for P. cyrtonema transcriptome assembly. Fig. S4A and Fig. S4B. (A) Venn diagram of annotated unigenes from the different databases. (B) Species distribution annotated in the NR database for P. cyrtonema. Fig. S5. GO function annotation of P. cyrtonema transcriptome. Fig. S6, Table S3. KEGG functional classifications of the annotated unigenes in P. cyrtonema. KEGG annotation of all unigenes. Table S4. UDP glycosyltransferases annotated in P. cyrtonema. Fig. S7. GOSlim analysis of rhizome-specifc up-regulation genes. Fig. S8. KEGG functional classifications of the rhizome-specifc up-regulation genes in P. cyrtonema. [file 13007_2019_441_MOESM1_ESM.docx]

**Transcriptome analysis of** ***Polygonatum cyrtonema* Hua: Identification of genes involved in polysaccharide biosynthesis**

Chenkai Wang^1,3^, Daiyin Peng^1,5^, Jinhang Zhu^2^, Derrui Zhao^1,3^, Yuanyuan Shi^1,3^, Shengxiang Zhang^1,3^, Kelong Ma^1,4^, Jiawen Wu^1,3,5*^, Luqi Huang^1,6*^.

^1^Anhui University of Chinese Medicine and Anhui Academy of Chinese Medicine, Hefei 230038, China.

^2^Anhui Medical University, Hefei 230032, China.

^3^Key Laboratory of Xin'an Medicine, Ministry of Education, Anhui University of Chinese Medicine, Hefei 230038, China. ^4^Clinical College of Integrated Traditional Chinese and Western Medicine, Anhui University of Chinese Medicine.

^5^Synergetic Innovation Center of Anhui Authentic Chinese Medicine Quality Improvement, Hefei 230012, China.

^6^State Key Laboratory Breeding Base of Dao-di Herbs，National Resource Center for Chinese Materia Medica, China Academy of Chinese Medical Sciences, Beijing China.
*Corresponding author. Jiawen Wu, Luqi Huang, E-mail address: wujiawen@ahtcm.edu.cn, huangluqi01@126.com.


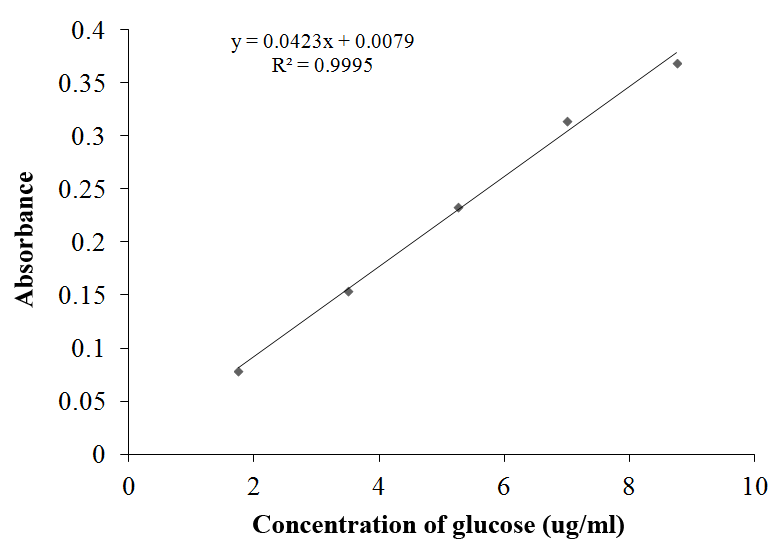


**Additional Figure 1. Standard curve of glucose at 582nm.**


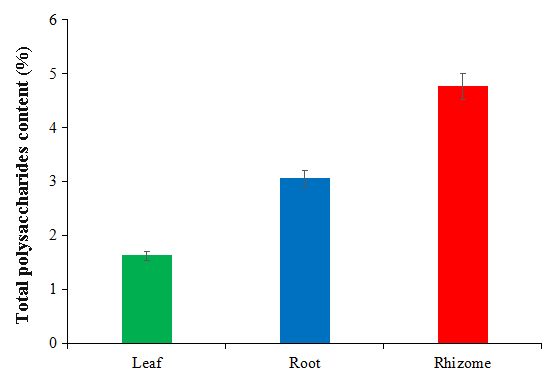


**Additional Figure 2. Total polysaccharides content from rhizomes, roots and leaves of *P. cyrtonema.***


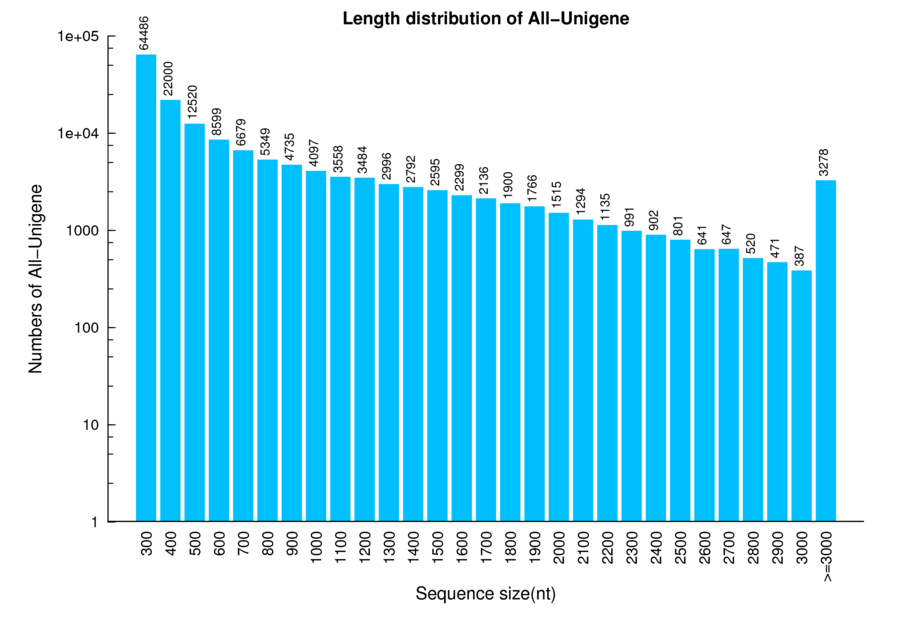


**Additional Figure 3. The length distribution of unigenes for** ***P. cyrtonema* transcriptome assembly.**


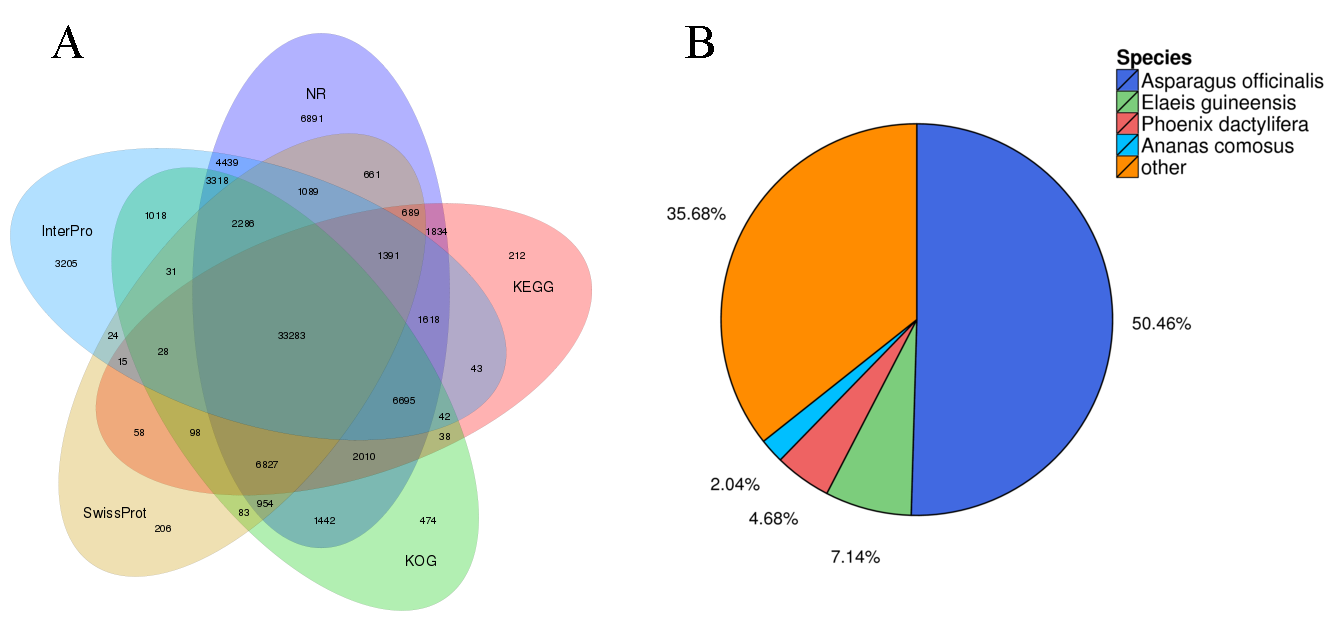


**Additional Figure 4. (A) Venn diagram of annotated unigenes from the different databases. (B) Species distribution annotated in the NR database for *P. cyrtonema.***


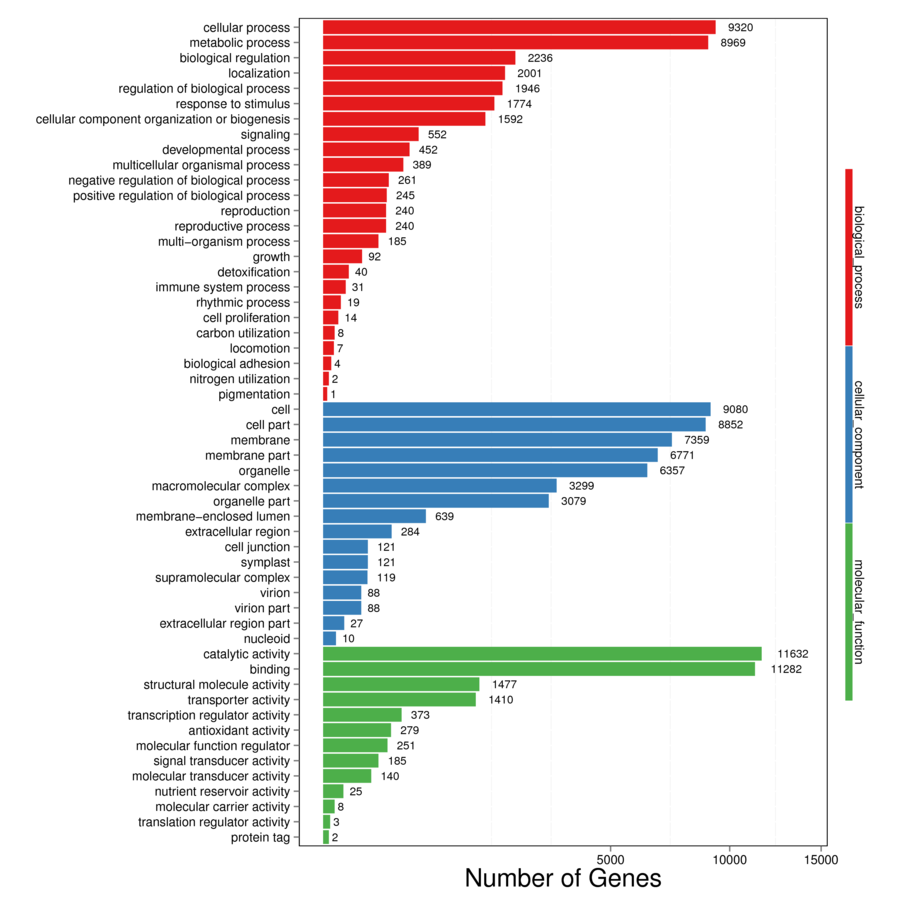
 **Additional Figure 5. GO function annotation of *P. cyrtonema* transcriptome.**


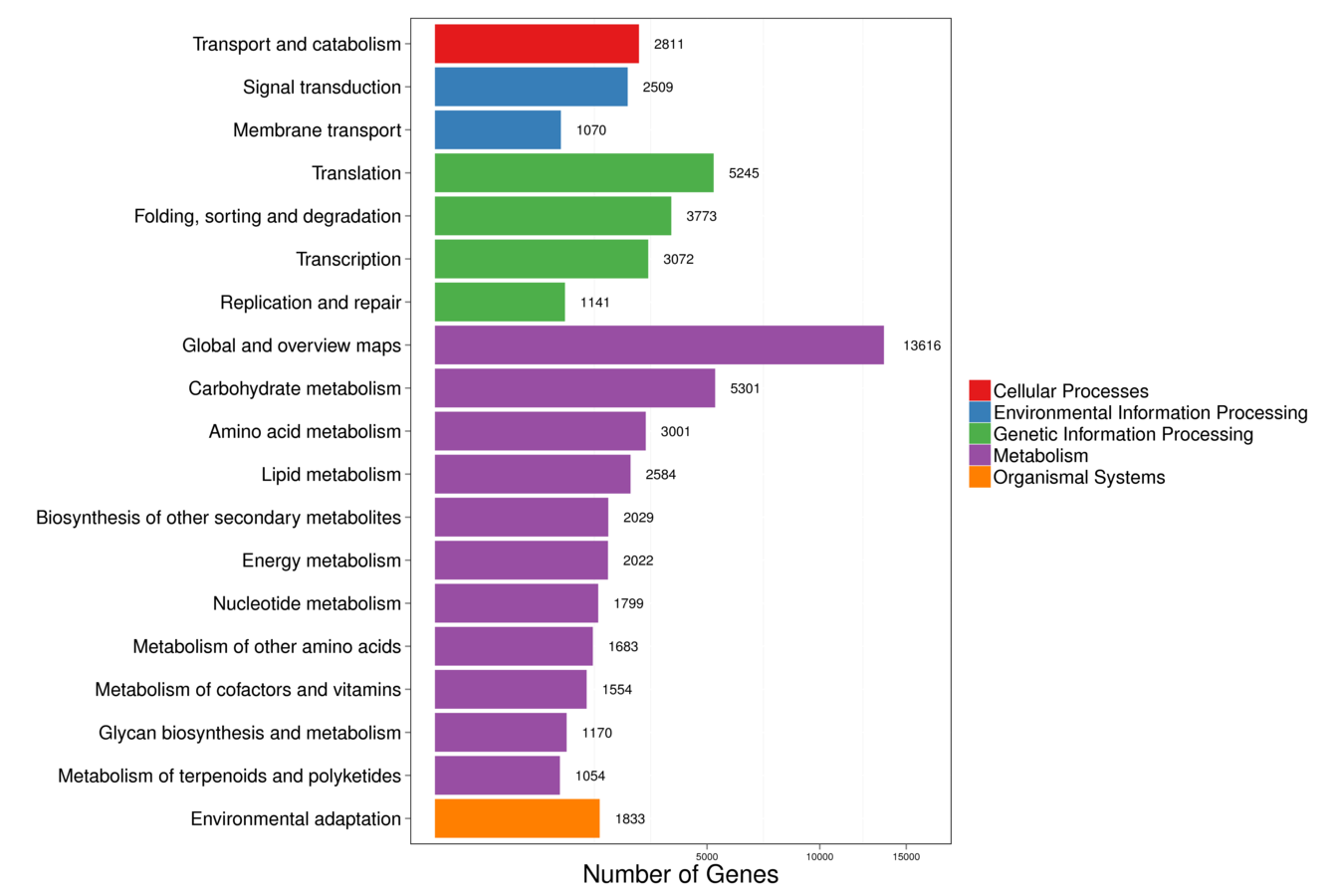
 **Additional Figure 6. KEGG functional classifications of the annotated unigenes in *P. cyrtonema*.**

**
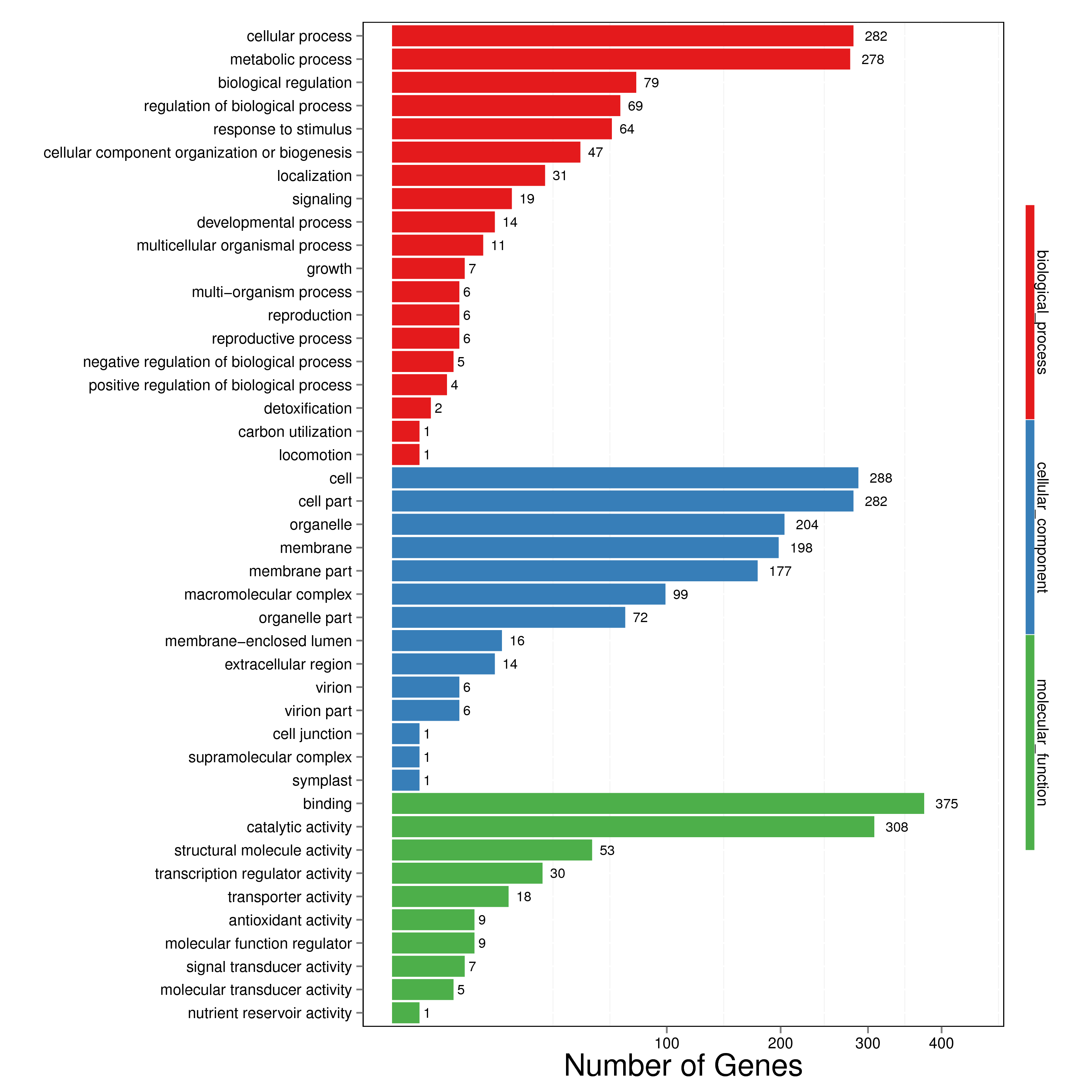
**

**Additional Figure 7. GOSlim analysis of rhizome-specifc up-regulation genes.**


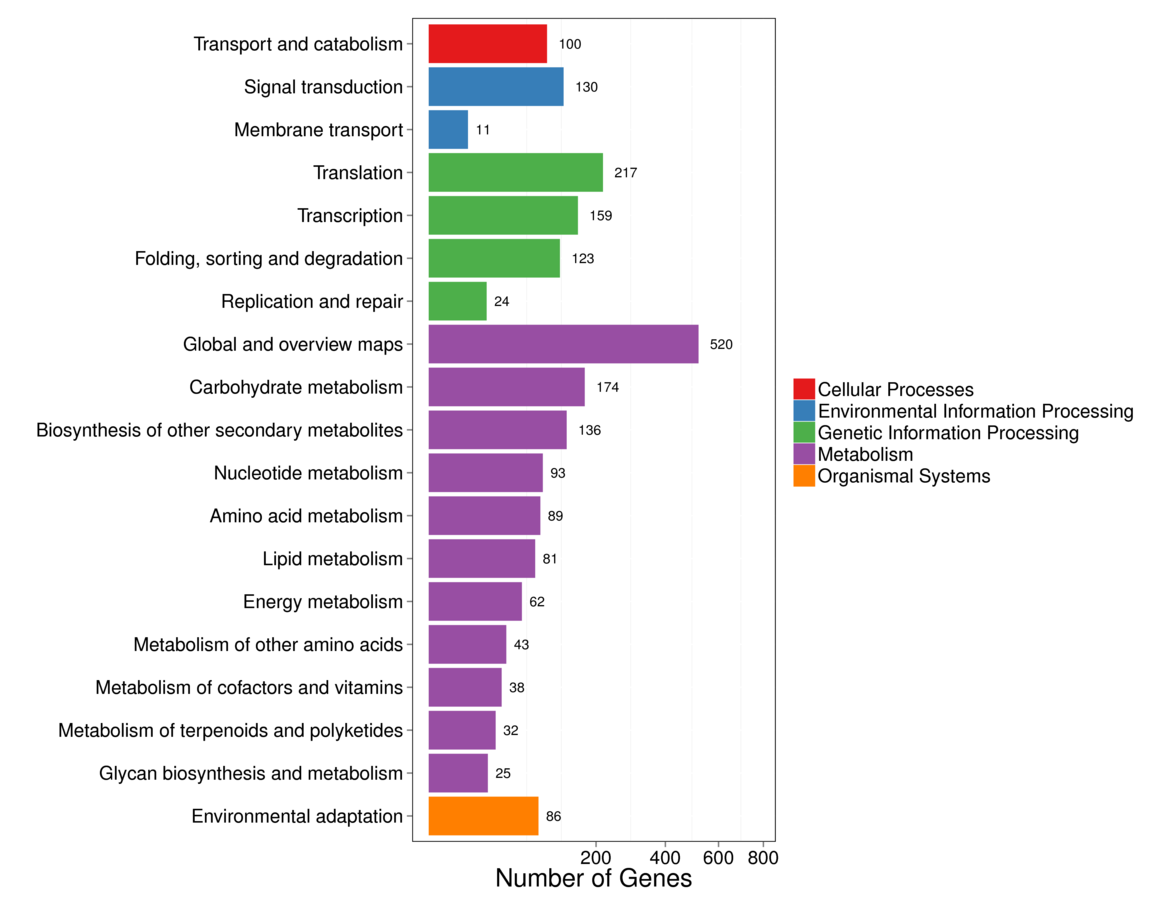


**Additional Figure 8. KEGG functional classifications of the** **rhizome-specifc up-regulation genes in *P. cyrtonema*.**

**Additional Table 1. RNA information of different tissues.**

| Sample | Concentration (ng/μL) | Total amount (μg) | OD260/280 | RIN | 28S/18S |
| --- | --- | --- | --- | --- | --- |
| Leaves | 208 | 4.16 | 1.91 | 7.3 | 1.7 |
| Roots | 360 | 7.2 | 1.89 | 7.9 | 3.1 |
| Rhizomes | 432 | 8.64 | 1.97 | 7.8 | 1.5 |

**Additional Table 2. Gene descriptions and primers used for qRT-PCR.**

| Genes | Amplicon Size （bp） | Primer pairs | TM value |
| --- | --- | --- | --- |
| *actin* | 103 | 5'-GGTCTATCTGGGAGGTGCAG-3' | 57.7 |
|  |  | 5'-GCTCAGACAGTTGAGTCCCT-3' | 56.8 |
| CL16751.Contig2 | 139 | 5'-AAGCAGAGAACGGTGATTGC-3' | 55.7 |
|  |  | 5'-CATCATCGGCATGCTTGACA-3' | 55.8 |
| CL3476. Contig4 | 97 | 5'-GCTATGAGAGGAAGGGCAGT-3' | 56.8 |
|  |  | 5'-CTCCGGTAGCGTTGTTGAAG-3' | 56.3 |
| CL3610.Contig2 | 139 | 5'-GACTGGGACGATCACCGTAT-3' | 56.6 |
|  |  | 5'-CCATTTGACGAGGAGAGGGT-3' | 56.9 |
| Unigene20408 | 126 | 5'-AAGGACAATGGCTGGGTACA-3' | 56.3 |
|  |  | 5'-TAGATGCGGCTGAACTGTCA-3' | 56.1 |

**Additional Table 3. KEGG annotation of all unigenes.**

| **Pathway** | **Unigene number** | **Pathway ID** |
| --- | --- | --- |
| Metabolic pathways | 12922 (23.55%) | ko01100 |
| Biosynthesis of secondary metabolites | 6979 (12.72%) | ko01110 |
| Carbon metabolism | 1971 (3.59%) | ko01200 |
| Ribosome | 1874 (3.41%) | ko03010 |
| Spliceosome | 1806 (3.29%) | ko03040 |
| RNA transport | 1800 (3.28%) | ko03013 |
| Biosynthesis of amino acids | 1679 (3.06%) | ko01230 |
| Protein processing in endoplasmic reticulum | 1647 (3%) | ko04141 |
| Endocytosis | 1643 (2.99%) | ko04144 |
| Purine metabolism | 1498 (2.73%) | ko00230 |
| Plant-pathogen interaction | 1480 (2.7%) | ko04626 |
| Pyrimidine metabolism | 1433 (2.61%) | ko00240 |
| Phenylpropanoid biosynthesis | 1402 (2.55%) | ko00940 |
| MAPK signaling pathway - plant | 1313 (2.39%) | ko04016 |
| Plant hormone signal transduction | 1200 (2.19%) | ko04075 |
| mRNA surveillance pathway | 1125 (2.05%) | ko03015 |
| Amino sugar and nucleotide sugar metabolism | 1078 (1.96%) | ko00520 |
| ABC transporters | 1070 (1.95%) | ko02010 |
| Starch and sucrose metabolism | 1038 (1.89%) | ko00500 |
| RNA polymerase | 969 (1.77%) | ko03020 |
| Ubiquitin mediated proteolysis | 967 (1.76%) | ko04120 |
| Glycolysis / Gluconeogenesis | 866 (1.58%) | ko00010 |
| Oxidative phosphorylation | 837 (1.53%) | ko00190 |
| RNA degradation | 830 (1.51%) | ko03018 |
| Pentose and glucuronate interconversions | 771 (1.4%) | ko00040 |
| Pyruvate metabolism | 725 (1.32%) | ko00620 |
| Glyoxylate and dicarboxylate metabolism | 718 (1.31%) | ko00630 |
| Galactose metabolism | 678 (1.24%) | ko00052 |
| Peroxisome | 658 (1.2%) | ko04146 |
| Citrate cycle (TCA cycle) | 645 (1.18%) | ko00020 |
| Cysteine and methionine metabolism | 619 (1.13%) | ko00270 |
| Ribosome biogenesis in eukaryotes | 615 (1.12%) | ko03008 |
| Fatty acid metabolism | 592 (1.08%) | ko01212 |
| Carbon fixation in photosynthetic organisms | 581 (1.06%) | ko00710 |
| 2-Oxocarboxylic acid metabolism | 576 (1.05%) | ko01210 |
| Phagosome | 547 (1%) | ko04145 |
| Glycerophospholipid metabolism | 543 (0.99%) | ko00564 |
| Other glycan degradation | 534 (0.97%) | ko00511 |
| Ascorbate and aldarate metabolism | 525 (0.96%) | ko00053 |
| Glycerolipid metabolism | 523 (0.95%) | ko00561 |
| Glutathione metabolism | 515 (0.94%) | ko00480 |
| Homologous recombination | 502 (0.91%) | ko03440 |
| Valine, leucine and isoleucine degradation | 483 (0.88%) | ko00280 |
| Aminoacyl-tRNA biosynthesis | 474 (0.86%) | ko00970 |
| Cyanoamino acid metabolism | 446 (0.81%) | ko00460 |
| Glycine, serine and threonine metabolism | 427 (0.78%) | ko00260 |
| Phosphatidylinositol signaling system | 406 (0.74%) | ko04070 |
| Alanine, aspartate and glutamate metabolism | 402 (0.73%) | ko00250 |
| DNA replication | 383 (0.7%) | ko03030 |
| Selenocompound metabolism | 380 (0.69%) | ko00450 |
| Inositol phosphate metabolism | 373 (0.68%) | ko00562 |
| Arginine and proline metabolism | 362 (0.66%) | ko00330 |
| Circadian rhythm - plant | 353 (0.64%) | ko04712 |
| Lysine degradation | 346 (0.63%) | ko00310 |
| Pentose phosphate pathway | 342 (0.62%) | ko00030 |
| Fatty acid degradation | 338 (0.62%) | ko00071 |
| Fructose and mannose metabolism | 338 (0.62%) | ko00051 |
| Propanoate metabolism | 336 (0.61%) | ko00640 |
| beta-Alanine metabolism | 333 (0.61%) | ko00410 |
| Arginine biosynthesis | 331 (0.6%) | ko00220 |
| Terpenoid backbone biosynthesis | 331 (0.6%) | ko00900 |
| Basal transcription factors | 329 (0.6%) | ko03022 |
| Tryptophan metabolism | 319 (0.58%) | ko00380 |
| Nucleotide excision repair | 317 (0.58%) | ko03420 |
| N-Glycan biosynthesis | 293 (0.53%) | ko00510 |
| Sphingolipid metabolism | 287 (0.52%) | ko00600 |
| Fatty acid biosynthesis | 283 (0.52%) | ko00061 |
| alpha-Linolenic acid metabolism | 272 (0.5%) | ko00592 |
| Nicotinate and nicotinamide metabolism | 271 (0.49%) | ko00760 |
| Proteasome | 267 (0.49%) | ko03050 |
| Carotenoid biosynthesis | 260 (0.47%) | ko00906 |
| Tyrosine metabolism | 254 (0.46%) | ko00350 |
| Nitrogen metabolism | 250 (0.46%) | ko00910 |
| Protein export | 241 (0.44%) | ko03060 |
| Porphyrin and chlorophyll metabolism | 233 (0.42%) | ko00860 |
| Phenylalanine, tyrosine and tryptophan biosynthesis | 230 (0.42%) | ko00400 |
| Mismatch repair | 228 (0.42%) | ko03430 |
| Biosynthesis of unsaturated fatty acids | 224 (0.41%) | ko01040 |
| Phenylalanine metabolism | 222 (0.4%) | ko00360 |
| Ubiquinone and other terpenoid-quinone biosynthesis | 214 (0.39%) | ko00130 |
| Flavonoid biosynthesis | 207 (0.38%) | ko00941 |
| Base excision repair | 202 (0.37%) | ko03410 |
| Ether lipid metabolism | 199 (0.36%) | ko00565 |
| Butanoate metabolism | 197 (0.36%) | ko00650 |
| Sulfur metabolism | 189 (0.34%) | ko00920 |
| Steroid biosynthesis | 187 (0.34%) | ko00100 |
| Fatty acid elongation | 183 (0.33%) | ko00062 |
| Pantothenate and CoA biosynthesis | 180 (0.33%) | ko00770 |
| Glycosaminoglycan degradation | 166 (0.3%) | ko00531 |
| Histidine metabolism | 164 (0.3%) | ko00340 |
| Glycosylphosphatidylinositol (GPI)-anchor biosynthesis | 161 (0.29%) | ko00563 |
| Cutin, suberine and wax biosynthesis | 153 (0.28%) | ko00073 |
| Valine, leucine and isoleucine biosynthesis | 152 (0.28%) | ko00290 |
| Photosynthesis | 150 (0.27%) | ko00195 |
| SNARE interactions in vesicular transport | 149 (0.27%) | ko04130 |
| Linoleic acid metabolism | 147 (0.27%) | ko00591 |
| Arachidonic acid metabolism | 147 (0.27%) | ko00590 |
| Limonene and pinene degradation | 144 (0.26%) | ko00903 |
| Isoquinoline alkaloid biosynthesis | 143 (0.26%) | ko00950 |
| One carbon pool by folate | 143 (0.26%) | ko00670 |
| Stilbenoid, diarylheptanoid and gingerol biosynthesis | 141 (0.26%) | ko00945 |
| Tropane, piperidine and pyridine alkaloid biosynthesis | 140 (0.26%) | ko00960 |
| Glycosphingolipid biosynthesis - ganglio series | 133 (0.24%) | ko00604 |
| Diterpenoid biosynthesis | 133 (0.24%) | ko00904 |
| Folate biosynthesis | 119 (0.22%) | ko00790 |
| Thiamine metabolism | 115 (0.21%) | ko00730 |
| Other types of O-glycan biosynthesis | 112 (0.2%) | ko00514 |
| Biotin metabolism | 107 (0.19%) | ko00780 |
| Lysine biosynthesis | 76 (0.14%) | ko00300 |
| Monobactam biosynthesis | 73 (0.13%) | ko00261 |
| C5-Branched dibasic acid metabolism | 73 (0.13%) | ko00660 |
| Vitamin B6 metabolism | 72 (0.13%) | ko00750 |
| Isoflavonoid biosynthesis | 71 (0.13%) | ko00943 |
| Indole alkaloid biosynthesis | 71 (0.13%) | ko00901 |
| Sesquiterpenoid and triterpenoid biosynthesis | 67 (0.12%) | ko00909 |
| Riboflavin metabolism | 63 (0.11%) | ko00740 |
| Taurine and hypotaurine metabolism | 62 (0.11%) | ko00430 |
| Brassinosteroid biosynthesis | 62 (0.11%) | ko00905 |
| Zeatin biosynthesis | 55 (0.1%) | ko00908 |
| Lipoic acid metabolism | 51 (0.09%) | ko00785 |
| Flavone and flavonol biosynthesis | 50 (0.09%) | ko00944 |
| Synthesis and degradation of ketone bodies | 48 (0.09%) | ko00072 |
| Sulfur relay system | 47 (0.09%) | ko04122 |
| Photosynthesis - antenna proteins | 46 (0.08%) | ko00196 |
| Phosphonate and phosphinate metabolism | 44 (0.08%) | ko00440 |
| Non-homologous end-joining | 44 (0.08%) | ko03450 |
| Glycosphingolipid biosynthesis - globo and isoglobo series | 35 (0.06%) | ko00603 |
| Monoterpenoid biosynthesis | 29 (0.05%) | ko00902 |
| Benzoxazinoid biosynthesis | 23 (0.04%) | ko00402 |
| Mannose type O-glycan biosynthesis | 19 (0.03%) | ko00515 |
| Anthocyanin biosynthesis | 19 (0.03%) | ko00942 |
| Caffeine metabolism | 18 (0.03%) | ko00232 |
| Betalain biosynthesis | 11 (0.02%) | ko00965 |
| Glucosinolate biosynthesis | 8 (0.01%) | ko00966 |
| Glycosphingolipid biosynthesis - lacto and neolacto series | 2 (0%) | ko00601 |
| D-Arginine and D-ornithine metabolism | 2 (0%) | ko00472 |

**Additional Table 4. UDP glycosyltransferases annotated in *P. cyrtonema*.**

| Query_id | Subject_id | Identity | E_value | Subject_annotation |
| --- | --- | --- | --- | --- |
| CL571.Contig2 | OVA01454.1 | 100 | 1.10E-04 | UDP-glucuronosyl/UDP-glucosyltransferase [Macleaya cordata] |
| CL571.Contig3 | OVA01454.1 | 72.41 | 1.60E-16 | UDP-glucuronosyl/UDP-glucosyltransferase [Macleaya cordata] |
| CL571.Contig5 | OVA01454.1 | 60.19 | 2.00E-28 | UDP-glucuronosyl/UDP-glucosyltransferase [Macleaya cordata] |
| CL1513.Contig1 | KVI08639.1 | 57.14 | 3.90E-20 | UDP-glucuronosyl/UDP-glucosyltransferase [Cynara cardunculus var. scolymus] |
| CL1513.Contig2 | XP_020269536.1 | 68.7 | 1.40E-41 | Putative UDP-rhamnose: rhamnosyltransferase 1 [Asparagus officinalis] |
| CL1513.Contig3 | XP_010910495.1 | 57.36 | 9.00E-148 | Putative UDP-rhamnose: rhamnosyltransferase 1 [Elaeis guineensis] |
| CL1513.Contig4 | XP_010910495.1 | 57.3 | 2.20E-149 | PREDICTED: putative UDP-rhamnose:rhamnosyltransferase 1 [Elaeis guineensis] |
| CL7256.Contig1 | OVA18489.1 | 49.14 | 2.10E-27 | UDP-glucuronosyl/UDP-glucosyltransferase [Macleaya cordata] |
| CL9379.Contig2 | OTF96907.1 | 64.29 | 6.70E-21 | putative UDP-glucuronosyl/ UDP-glucosyltransferase [Helianthus annuus] |
| CL9728.Contig1 | XP_010943217.1 | 66.52 | 6.80E-179 | PREDICTED: limonoid UDP-glucosyltransferase [Elaeis guineensis] |
| CL9728.Contig2 | XP_010943217.1 | 66.95 | 6.50E-180 | PREDICTED: limonoid UDP-glucosyltransferase [Elaeis guineensis] |
| CL9728.Contig3 | XP_010943217.1 | 66.52 | 5.30E-179 | PREDICTED: limonoid UDP-glucosyltransferase [Elaeis guineensis] |
| CL12526.Contig2 | AIF76151.1 | 57.23 | 4.60E-162 | UDP-glucosyltransferase UGT85U2, partial [Crocus sativus] |
| CL14495.Contig1 | OVA19028.1 | 37.14 | 3.00E-41 | UDP-glucuronosyl/UDP-glucosyltransferase [Macleaya cordata] |
| CL14495.Contig2 | OVA19028.1 | 40.51 | 5.50E-72 | UDP-glucuronosyl/UDP-glucosyltransferase [Macleaya cordata] |
| CL16103.Contig1 | OVA18489.1 | 48.45 | 6.80E-121 | UDP-glucuronosyl/UDP-glucosyltransferase [Macleaya cordata] |
| CL16103.Contig2 | OVA18489.1 | 49.47 | 8.10E-121 | UDP-glucuronosyl/UDP-glucosyltransferase [Macleaya cordata] |
| CL16787.Contig1 | AIF76151.1 | 53.25 | 1.20E-149 | UDP-glucosyltransferase UGT85U2, partial [Crocus sativus] |
| Unigene14962 | XP_009413144.1 | 65.38 | 1.90E-175 | PREDICTED: UDP-glycosyltransferase 86A1 [Musa acuminata subsp. malaccensis] |
| Unigene19319 | OVA18815.1 | 52.86 | 1.30E-155 | UDP-glucuronosyl/UDP-glucosyltransferase [Macleaya cordata] |
| Unigene23260 | OVA18815.1 | 55.74 | 1.10E-159 | UDP-glucuronosyl/UDP-glucosyltransferase [Macleaya cordata] |
| Unigene28535 | AIF76150.1 | 73.53 | 7.50E-05 | UDP-glucosyltransferase UGT85V1 [Crocus sativus] |
| Unigene30344 | AIF76151.1 | 60.4 | 2.00E-91 | UDP-glucosyltransferase UGT85U2, partial [Crocus sativus] |
| Unigene80963 | BAO51837.1 | 40.95 | 3.40E-36 | UDP-glycosyltransferase 73A17 [Camellia sinensis] |
| Unigene86842 | AIF76152.1 | 73.97 | 3.30E-26 | UDP-glucosyltransferase UGT85U1 [Crocus sativus] |
| Unigene89041 | OVA19029.1 | 48.45 | 1.40E-16 | UDP-glucuronosyl/UDP-glucosyltransferase [Macleaya cordata] |
| Unigene106072 | XP_010943278.1 | 60.22 | 1.30E-27 | PREDICTED: limonoid UDP -glucosyltransferase-like [Elaeis guineensis] |
